# Direct Current Stimulation (DCS) Modulates Lipid Metabolism and Intercellular Vesicular Trafficking in SHSY‐5Y Cell Line: Implications for Parkinson's Disease

**DOI:** 10.1111/jnc.70014

**Published:** 2025-02-10

**Authors:** Marco Piccoli, Luisa Barbato, Natale Vincenzo Maiorana, Alessandra Mingione, Francesca Raimondo, Marco Ghirimoldi, Federica Cirillo, Mattia Schiepati, Domenico Salerno, Luigi Anastasia, Elisabetta Albi, Marcello Manfredi, Tommaso Bocci, Alberto Priori, Paola Signorelli

**Affiliations:** ^1^ Institute for Molecular and Translational Cardiology (IMTC) IRCCS Policlinico San Donato Milan Italy; ^2^ School of Medicine University Vita‐Salute San Raffaele Milan Italy; ^3^ Biochemistry Laboratory IRCCS Policlinico San Donato Milan Italy; ^4^ “Aldo Ravelli” Research Centre, Department of Health Sciences University of Milan Milan Italy; ^5^ School of Medicine and Surgery University of Milan‐Bicocca Monza Italy; ^6^ Biological Mass Spectrometry Lab, Department of Translational Medicine University of Piemonte Orientale Novara Italy; ^7^ School of Medicine and Surgery BioNanoMedicine Center NANOMIB University of Milan‐Bicocca Monza Italy; ^8^ Department of Pharmaceutical Sciences, Interno Orto Botanico University of Perugia Perugia Italy; ^9^ Center for Translational Research Autoimmune Diseases and Allergic Diseases University of Piemonte Orientale Novara Italy

**Keywords:** direct current stimulation (DCS), extracellular vesicles, inflammation, lipids, neurodegeneration, neuroplasticity

## Abstract

The modulation of the brain's electrical activity for therapeutic purposes has recently gained attention, supported by the promising results obtained through the non‐invasive application of transcranial direct current stimulation (tDCS) in the treatment of neurodegenerative and neurological diseases. To optimize therapeutic efficacy, it is crucial to investigate the cellular and molecular effects of tDCS. This will help to identify important biomarkers, predict patient's response and develop personalized treatments. In this study, we applied direct current stimulation (DCS) to a neural cell line, using mild currents over short periods of time (0.5 mA, 20 min), with 24‐h intervals. We observed that DCS induced changes in the cellular lipidome, with transient effects observed after a single stimulation (lasting 24 h) and more significant, long‐lasting effects (up to 72 h) after repeated stimulation cycles. In neural cells, multiple DCS treatment modulated structural membrane lipids (PE, PS, PI), downregulated glycerol lipids with ether‐linked fatty acids and pro‐inflammatory lipids (ceramides and lyso‐glycerophospholipids) (*p* ≤ 0.005). Multiple DCS sessions altered transcriptional activity by decreasing the expression of inflammatory cytokines (TNF‐α, *p* ≤ 0.05; IL‐1β, *p* ≤ 0.01), while increasing the expression of neuroprotective factors such as heme oxygenase‐1 (*p* ≤ 0.0001) and brain‐derived neurotrophic factor (*p* ≤ 0.05), as well as proteins involved in vesicular transport (SNARE, sorting nexins and seipin and α‐synuclein; *p* ≤ 0.05). In addition, DCS enhanced the release of extracellular vesicles, with repeated stimulations significantly increasing the release of exosomes threefold. In conclusion, while a single electrical stimulation induces transient metabolic changes with limited phenotypic effects, repeated applications induce a broader and deeper modulation of lipid species. This may lead to a neuroprotective and neuroplasticity‐focussed transcriptional profile, potentially supporting the therapeutic effects of tDCS at the cellular and molecular level in patients..

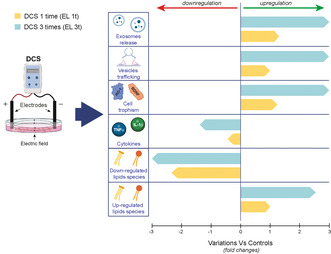

## Graphical abstract

Direct current stimulation (DCS) is considered a suitable treatment for neurodegenerative diseases. This study investigates how DCS affects lipid metabolism and signaling in neuroblastoma cells. A single DCS session leads to transient metabolic changes, while repeated stimulations show lasting effects. Repeated DCS downregulates pro‐inflammatory lipids and cytokines and upregulates neuroprotective factors. Multiple stimulations also enhanced vesicular release, including a threefold increase in exosome secretion, supporting neuronal neuroplasticity. Lipidomic analysis revealed membrane remodeling, a reduction in proinflammatory lipids, and increased fatty acid oxidation. These results suggest that DCS may be neuroprotective, emphasizing its potential as a therapeutic tool for neurodegenerative diseases.

**Abbreviations**
ACADL: long chain acyl‐CoA dehydrogenase
ACADM: medium chain acyl‐CoA dehydrogenases
BDNF: brain‐derived neurotrophic factor
BSLC2: lipid droplet biogenesis associated
CAR: acylcarnitine
Cer: ceramide
CPT1a: carnitine palmitoyl transferase 1a
CPT1c: carnitine palmitoyl transferase 1c
DAG‐O: ether‐linked diglyceride
DG: diglyceride
DRL: differentially regulated lipid
FA: fatty acid
HexCer: monoglycosylated ceramide
HO‐1: heme oxygenase‐1
IL‐1β: interleukin‐1 beta
LCMS/MS: liquid chromatography‐mass spectrometry
L EVs: large extracellular vesicles
LPC: lysophosphatidylcholine
LPE: lysophosphatidylethanolamine
LTD: long‐term depression
LTP: long‐term potentiation
LysoGL: lysoglycerolipid
NAE: *N*‐acylethanolamine
PC: phosphatidylcholine
PCA: principal component analysis
PC‐O: ether‐linked phosphatidylcholine
PE: phosphatidylethanolamine
PE‐O: ether‐linked phosphatidylethanolamine
PI: phosphatidylinositol
PS: phosphatidylserine
RRID: Research Resource Identifier (see scicrunch.org)
S EVs: small extracellular vesicles
SM: sphingomyelin
SNAP25: synaptosome associated protein
SNARE: SNAP receptor
SNCA: α‐synuclein
SNX14: sorting nexin 14
STX1A: syntaxin 1A
tDCS: transcranial direct current system
TG: triglyceride
TNF‐α: tumor necrosis factor

## 1 Introduction

Modulation of the brain's electrical activity has been shown to be a potential therapeutic approach for various pathological conditions. Transcranial direct current stimulation (tDCS) is a non‐invasive neuromodulatory technique in which a continuous low‐intensity electrical current (typically between 1 and 2 mA) is applied via electrodes on the scalp (Priori et al. 1998; Lefaucheur et al. 2017; Woods et al. 2016). The current flows between an anode and a cathode placed on specific regions of the scalp and alters the membrane potential of the neurons in the relevant cortical area. This leads to changes in cortical excitability and modulates synaptic plasticity. tDCS can either excite (with anodal stimulation) or inhibit (with cathodal stimulation) brain activity in the affected areas (Antal et al. 2003; Nitsche and Paulus 2000).

Due to its therapeutic potential, tDCS is gaining increasing attention in the clinical field. It has been used in the treatment of various neurological and psychiatric disorders, including major depressive disorder, where it shows antidepressant effects (Aparicio et al. 2016; D'Urso et al. 2022), and in stroke rehabilitation, where it supports motor recovery (Bornheim et al. 2022). In addition, tDCS has been investigated for the treatment of chronic pain, as it can modulate pain perception by altering activity in cortical areas associated with pain (Lefaucheur et al. 2008). Its role in neurodegenerative diseases, such as Alzheimer's and Parkinson's, is also being investigated. There is evidence that it can modulate neuroplasticity and possibly slow cognitive decline (Suarez‐Garcia et al. 2020).

The effectiveness of tDCS depends on several factors, including the number of sessions, the duration, and the intensity of the current. Protocols must be carefully tailored to optimize therapeutic outcomes (Lefaucheur et al. 2017). To achieve this, understanding the mechanisms of tDCS at the systemic, cellular, and molecular levels is essential. This requires the identification of simple models and specific targets that can serve as therapeutic and prognostic markers in clinical practice. tDCS alters the firing rates of neurons by modulating their membrane potential (Nitsche and Paulus 2000). The effects of tDCS can persist over long periods of time, and the duration and magnitude of these effects are directly influenced by the intensity and duration of the applied current (Nitsche and Paulus 2001). Importantly, these effects can be enhanced when combined with training protocols that involve repeated cycles of stimulation (Laste et al. 2012). One area of particular interest is the effect of tDCS on ion channels and neuronal excitability. The electric fields generated by tDCS modulate voltage‐gated ion channels, affecting resting membrane potential and synaptic transmission, thereby impacting synaptic plasticity and neuronal function (Bikson et al. 2019; Vasu and Kaphzan 2023). Furthermore, tDCS influences the activity of neurotransmitter receptors, which play a key role in long‐term potentiation (LTP) and long‐term depression (LTD) (Kronberg et al. 2017; Xin et al. 2005).

In addition, electrical stimulation has been applied in a rat model of vascular dementia and has been shown to affect autophagy, a process known to be downregulated in response to various stressors and in neurodegenerative diseases (Guo et al. 2020).

DCS can also be applied to cell cultures (Mattioli et al. 2024). Depending on the materials used as electrodes, different combinations of effects have been observed (Zhu et al. 2019; Rabbani et al. 2024; Allioux et al. 2023). DCS activates signaling pathways in individual cells that regulate functions such as migration, alignment, or proliferation (Ariza et al. 2010; Martin et al. 2022; Chen et al. 2019). Electrical stimulation regulates events within the membrane‐cytosol‐nucleus, alters chromatin folding, and promotes extracellular communication (Karunasagara et al. 2025), the differentiation of neuronal cells, fibroblasts, osteoblasts, and different types of stem cells (embryonic, neuronal, and mesenchymal) in regenerative medicine and wound healing (Calzada, Onguka, and Claypool 2016; Chen et al. 2019; Martin et al. 2022). More recently, DCS has been shown to stimulate autophagy in neurons and favor the release of synucleopathy stress in a Parkinson's cell model (He et al. 2021; Sala et al. 2021).

The electrical and mechanical properties of membranes are inextricably linked to the composition of their lipid‐protein bilayer, whose charges are regulated by the energy input and propagate across the membrane. Dynamic structural changes lead to a constant asymmetry in the components, resulting in a negatively charged inner surface compared to the outer surface, creating the conditions for a force similar to piezoelectricity (Brownell, Qian, and Anvari 2010). Changes in the external electric field lead to rapid and profound changes in transmembrane potential by modulating components that are intrinsically connected to intracellular signaling via lipid metabolites and protein interactions and activities. Living cells adapt to stimuli through immediate membrane responses and have an intrinsic tendency to restore homeostasis. Therefore, the intensity, duration, and frequency of the stimulus are key factors that determine the cellular response. Nature teaches us that the membrane is equipped with molecules that contribute to the maintenance of cellular homeostasis when the external environment requires it, such as the increased concentration of saturated fatty acids in response to low temperatures (Wu, Baumeister, and Heimbucher 2023). Thus, electrical stimulation can induce changes in the membrane lipidome, starting from the plasma membrane, which is in direct communication with all cell membranes. These changes may result in either a transient reaction or an adaptive response.

In this study, we focus on the effects of DCS on lipid metabolism by performing a lipidomic analysis on neuroblastoma cells treated with direct current stimulation. By examining the changes in lipid profiles and comparing the differential effects between single and multiple stimulations, we aim to gain new insights into how DCS affects lipid metabolism at the cellular level, potentially contributing to its broader neurobiological and neuroprotective effects. These results may help to clarify the cellular mechanisms underlying tDCS and its therapeutic potential.

## 2 Materials and Methods

### 2.1 Cells and Treatments

SH‐SY5Y cells, a human neuroblastoma cell line that expresses neural stem cell and neuronal markers and derives from dopaminergic neurons, were grown in DMEM high glucose supplemented with 10% FBS, 1% penicillin/streptomycin, and 1% l‐glutamine, and incubated in a 5% CO_2_ humidified atmosphere at 37°C. SH‐SY5Y were used up to approximately passage 35/40 before thawing a new aliquot of cells.

This cell line is not listed as a commonly misidentified cell line by the International Cell Line Authentication Committee (ICLAC) and was provided by ATCC (RRID:CVCL_00199), who also guarantee the high‐quality cell authentication.

### 2.2 Direct Current Stimulation (DCS)

SH‐SY5Y were seeded at a density of 5 × 10^5^ in 60 mm cell culture dishes, and after 24 h, they were subjected to the stimulation protocol with 0.5 mA/mm^2^ direct current stimulation (DCS) for 20 min, with an initial current ramp of increasing intensity and a final ramp of 20 s at different time points (24, 72 h) and compared with non‐stimulated cells used as control. Stimulation was performed with two electrodes soaked in PBS and immersed in the cell culture medium in diametrically opposite positions and connected to a battery‐powered stimulator (HDCStim, Newronika). Prior to stimulation, culture medium (3.5 mL) was added to each dish to achieve a sufficient volume to ensure the correct flow of current. During stimulation, the cell dishes were kept without lids at 37°C in an atmosphere of 5% CO_2_ in air, while the stimulator was kept outside the incubator. For each experiment, controls were performed with a specific dish subjected to the stimulation protocol without current passage (Sala et al. 2021).

### 2.3 RNA Extraction and Real Time‐PCR

Total RNA was isolated from harvested cells using the ReliaPrep Miniprep RNA extraction system (Promega, Madison, WI, USA, cat. no. Z6011), according to the manufacturer's instructions. Then, 2 μg of the purified RNA was reverse transcribed into cDNA. Amplification was performed for the following genes: *IL‐1β*, *HO‐1*, *TNF‐α*, *BDNF*, *ACADM*, *ACADL*, *CPT1a*, *CPT1c*, *SNAP25*, *STX1A*, *BSCL2* (*SEIPIN*), *SNX14*, and *SNCA*; all the primer sequences are available in Table S1 of the Supporting Information. Relative mRNA of target genes was normalized to endogenous *GAPDH* gene expression and represented as fold change over unstimulated cells using the comparative Ct method (∆∆Ct methods). Real‐time PCR was performed using the GoTaq qPCR Master Mix (Promega, Madison, WI, USA, cat. no. A6002) following the manufacturer's instructions.

### 2.4 Cells Proliferation Analysis

SH‐SY5Y were seeded at a density of 5 × 10^5^ in 60 mm and exposed to the DCS protocol. Alive cells were counted after excluding trypan blue‐positive cells, after 24 and 72 h after the electrical stimulation. Cell number was determined by the automated cell counter Countess II FL (Life Technologies, Carlsbad, CA, USA, RRID:SCR_025370).

### 2.5 Apoptosis Analysis by Hoechst 33342 Staining

Electrostimulated cells at different time points were fixed in paraformaldehyde 4% for 15 min at room temperature (RT) and then washed 3 times with PBS. Cells were permeabilized by PBS with 0.1% Triton‐X100 and then incubated with PBS with 5% BSA + 0.1% Triton‐X100 for 15 min at RT. Successively, cells were stained with Hoechst 33342 (100 ng/mL in PBS) (Merck, Darmstadt, Germany, cat. no. 14533) for 15 min at RT. Apoptotic cells were observed under a fluorescent microscope (Olympus TH4‐200, Olympus Corporation, Shinjuku‐ku, Tokyo, Japan) with magnification 20×. The percentage of apoptotic cells was calculated by normalizing the number of stained nuclei to the number of total nuclei. The counts have been performed in 15 different fields for each sample.

### 2.6 Lipid Extraction

Lipids from electrostimulated cells at different time points were extracted using 1 mL of 75:25 isopropanol (IPA)/H_2_O solution using a one‐phase extraction after the addition of 100 μL of 5% CH_3_OH deuterated standard (Splash Lipidomix). One‐phase extraction is simpler to implement than Bligh & Dyer and Folch extractions, with comparable or better analytical performance (Calderon et al. 2019). Then the samples were vortexed for 30 s, sonicated for 2 min, vortexed again for 30 s, and then they were incubated for 30 min at 4°C under gentle, constant shaking. Subsequently, samples were rested on ice for an additional 30 min. Centrifugation for 10 min at 3500×*g* at 4°C was performed to remove debris and other impurities. 1 mL of extracted lipids was collected and dried using a SpeedVac centrifuge (Labogene). The dried samples were reconstituted in 100 μL of CH_3_OH containing the internal standard CUDA (12.5 ng/mL). After reconstitution, samples were analyzed with a Vanquish UHPLC system (Thermo Scientific, Rodano, Italy) coupled with an Orbitrap Q‐Exactive Plus (Thermo Scientific, Rodano, Italy). Lipids were separated by a reversed‐phase column (Hypersil Gold 150 × 2.1 mm, particle size 1.9 μm) maintained at 45°C with a flow rate of 0.260 mL/min. For electrospray ionization (ESI) positive mode, mobile phase A was obtained with a solution of 60:40 (v/v) acetonitrile/water with ammonium formate buffer (10 mM) and 0.1% formic acid, and a solution of 90:10 isopropanol/acetonitrile (v/v) with ammonium formate (10 mM) and 0.1% formic acid was used for mobile phase B. For the negative (ESI) mode, the same organic solvents for both mobile phases were used except for ammonium acetate (10 mM) as a mobile phase modifier. The gradient used was as follows: 0–2 min from 30% to 43% B, 2–2.1 min from 43% to 55% B, 2.1–12 min from 55% to 65% B, 12–18 min at 65% to 85% B, 18–20 min at 85% to 100% B; 100% B was held for 5 min, and then the column was allowed to equilibrate to 30% B for another 5 min. The total running time was 30 min. Mass spectrometry analysis was performed in both positive ion (at 3.5 kV) and negative ion (2.8 kV) modes. Data were collected in a data‐dependent top 10 scan mode (ddMS2). MS full‐scan survey spectra (mass range m/z 80–1200) were acquired with a resolution of *R* = 70000 and a target AGC of 1 × 10^6^. MS/MS fragmentation was performed using high‐energy c‐trap dissociation (HCD) with *R* = 17500 resolution and 1 × 10^5^ AGC target. The step normalized collision energy (NCE) was set to 15, 30, and 45. The injection volume was 3 μL. For accurate mass‐based analysis, regular Lockmass and interrun calibrations were used. An exclusion list for background ions was generated by testing the same procedural sample for both positive and negative ESI ionization modes. Quality control was ensured by analyzing pooled samples before, at the beginning, and at the end of the batches; using blanks to check for residual interference; and using internal standards, directly in plasma or cell samples, which include a series of analyte classes at levels appropriate for the plasma (Avanti SPLASH Lipidomix) and an internal standard (CUDA) prior to liquid chromatography‐mass spectrometry (LC–MS) analysis. Raw data acquired from lipidomic untargeted analysis were processed with MSDIAL software (Yokohama City, Kanagawa, Japan), version 4.24. Peaks were detected, MS2 data were deconvoluted, compounds were identified, and peaks were aligned across all samples. The peak areas for the different molecular species detected were normalized using the deuterated internal standard for each lipid class in order to obtain their quantitation. To obtain an estimated concentration expressed in nmol/mL (plasma), the normalized areas were multiplied by the concentration of the internal standard. An in‐house library of standards was also used for lipid identification.

### 2.7 Isolation of EVs From Electrostimulated Cells

For the isolation of EVs from cells, a medium supplemented with exosome‐depleted serum was used to avoid possible external contamination. Conditioned media from electrostimulated cells at different time points were first centrifuged at 500×*g* for 20 min RT to remove dead cells. EV isolation was performed by differential centrifugation and ultracentrifugation; all steps were performed at 4°C. In brief, the collected supernatants were first centrifuged at 2000×*g* for 20 min (Benchtop centrifuge 5804 R, Eppendorf), then at 10 000×*g* for 30 min (Avanti J‐25 Beckman, swing‐out rotor JS 13.1). The pellets (10K) were dissolved in PBS and stored at −80°C. The supernatant was transferred to clean tubes and ultracentrifuged at 200 000×*g* for 90 min (Optima L‐90K, Beckman, rotor type 50.2 Ti). After removing the supernatant, the pellets (200K) were collected in PBS, and stored at −80°C until use.

### 2.8 EVs Characterization: Nanoparticles Tracking Analysis and Western Blotting

The size distribution and concentration of EVs were measured by nanoparticle tracking analysis (NTA) using a NanoSight NS300 (Malvern Instrument Inc., Malvern, UK) equipped with a high‐sensitivity camera (Hamamatsu), an objective lens, a syringe pump system, and a 488 nm laser. The camera operated at 30 frames per second (fps). Prior to injection, the EVs were diluted in sterile PBS. The resulting tracking graphs were analyzed using NTA 3.2 software (dev build 3.2.16, Malvern Panalytical, Malvern, UK) with a threshold of 4.

EV proteins were separated using the NuPAGE electrophoresis system, using 4%–12% NuPAGE and MOPS (3‐[N‐morpholino] propane sulfonic acid) sodium dodecyl sulphate (SDS) buffer. Proteins were transferred to nitrocellulose membranes using an electrophoretic “tank” transfer device (Hoefer, Holliston, MA, USA) to detect typical markers for small EVs (TSG‐101 1:1000, TSG101 mouse monoclonal antibody [4A10], cat. no. MA1‐23296; Thermo Fisher Scientific, Waltham, MA, USA; Alix 1:2000, anti‐ALIX RabMab antibody [EPR15314], cat. no. ab186429, Abcam, Cambridge, UK).

### 2.9 Statistical Analysis

All statistical analyses were performed using GraphPad Prism 9.1.0 software (GraphPad Software, San Diego, CA, USA, GraphPad Prism; RRID:SCR_002798). The Shapiro–Wilk test was performed for each data set to assess the normality of the distribution, along with analysis to identify possible outliers by the ROUT method. All samples were normally distributed, and any data point was excluded from the analysis (Table S2). For experiments comparing two groups, a two‐tailed unpaired Student's *t*‐test was performed. Alternatively, when comparing three or more groups, an ordinary one‐way ANOVA was performed, followed by a Tukey's multiple comparison test with a single pooled variance. Unless otherwise stated, the columns of the histograms represent the mean ± SEM and were calculated from the experimental replicates. Data were considered statistically significant if *p* ≤ 0.05, as indicated in the figure legends and in the full statistical report (Table S3).

## 3 Results

**FIGURE 1:**
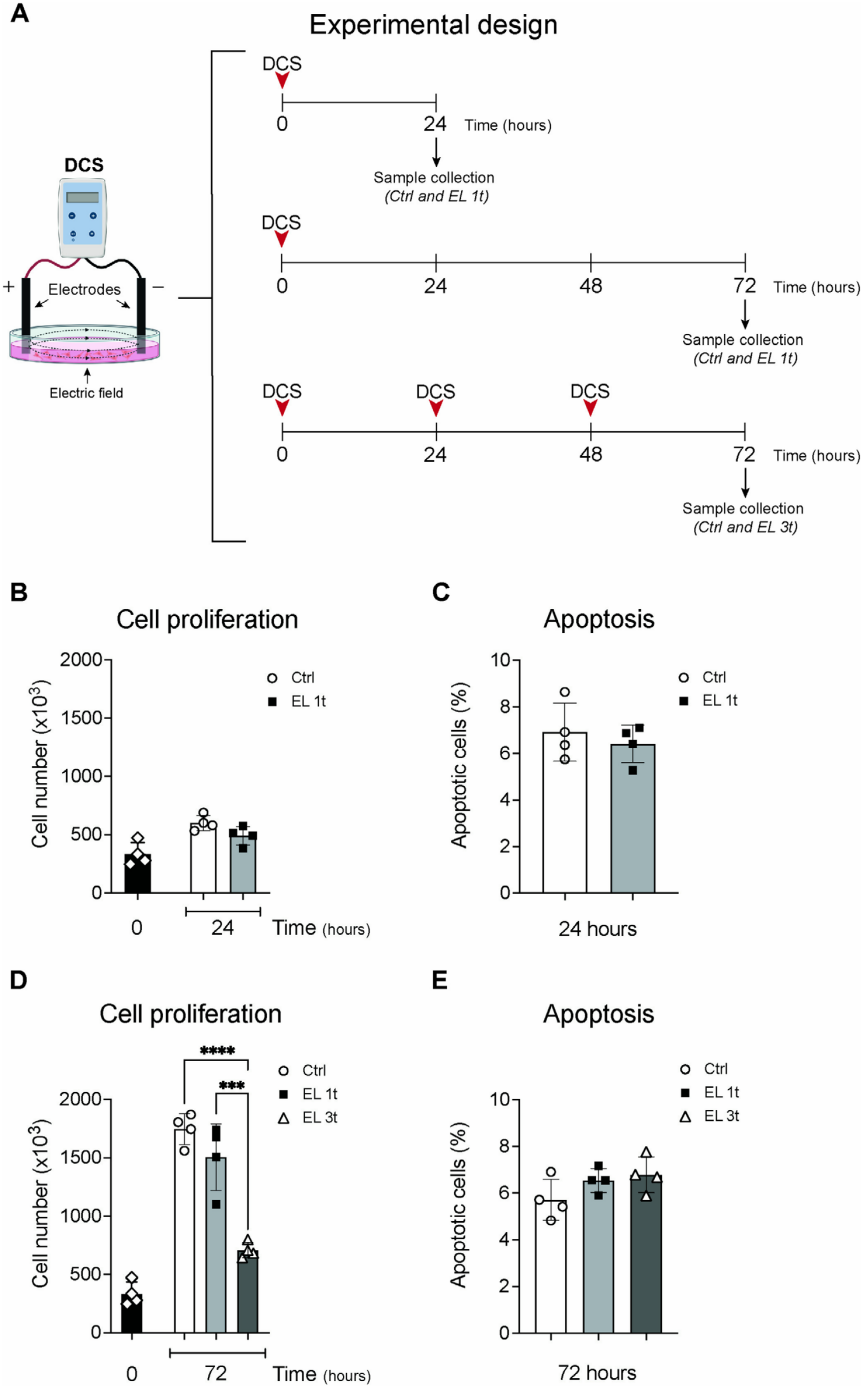
Effects of DCS on cell proliferation and apoptosis. Experimental design for DCS application and sample collection. In vitro model of DCS applied to SH‐SY5Y neuroblastoma cells (A). SH‐SY5Y proliferation (B) and apoptosis (C) 24 h after stimulation. SH‐SY5Y proliferation and (D) apoptosis (E) after 72 h. Data are expressed as cell number for proliferation analysis and percentage of apoptotic cells. Controls are represented by non‐electrically stimulated cells. Data are represented as mean ± SEM, and each point in the graphs represents an experimental replicate. Statistical significance was determined by a two‐tailed Student's *t*‐test or ordinary one‐way ANOVA. ***p < 0.001; ****p < 0.0001; *n* = 4.

A static electric field was applied to SH‐SY5Y neuroblastoma cell cultures, and the cells were stimulated with 0.5 mA direct current (DCS) for 20 min. The stimulated cells were divided into three experimental groups (Figure 1A):

- Single DCS stimulation “early effects” (upper panel): A single DCS session was administered at time 0, and samples were collected 24 h later for analysis (Ctrl and EL 1t groups; 24 h).
- Single DCS stimulation “late effects” (centre panel): A single DCS session was administered at time 0, and samples were collected 72 h later for analysis (Ctrl and EL 1t groups; 72 h).
- Three DCS stimulations (lower panel): Three DCS sessions were administered 24 h apart, with stimulations at time 0, 24, and 48 h. Samples were collected 72 h after the first stimulation (groups Ctrl and EL 3t; 72 h).

The effects of a single DCS stimulation (EL 1t) on cell proliferation and apoptosis were analyzed 24 and 72 h after stimulation. In addition, the effects of multiple DCS stimulations (EL 3t) were assessed 72 h after the first stimulation (Figure 1). At 24 h, there were no significant differences in cell number or percentage of apoptotic cells between the controls (not electrically stimulated cells) and the EL 1t group (Figure 1B,C). After 72 h, the survival and the proliferation rate were unaffected in cells subjected to a single stimulation. In contrast, multiple stimulations led to a significant reduction in proliferation compared to both the control group and the EL 1t group (Figure 1D), but no significant differences in apoptosis were observed after 72 h (Figure 1E).

**FIGURE 2:**
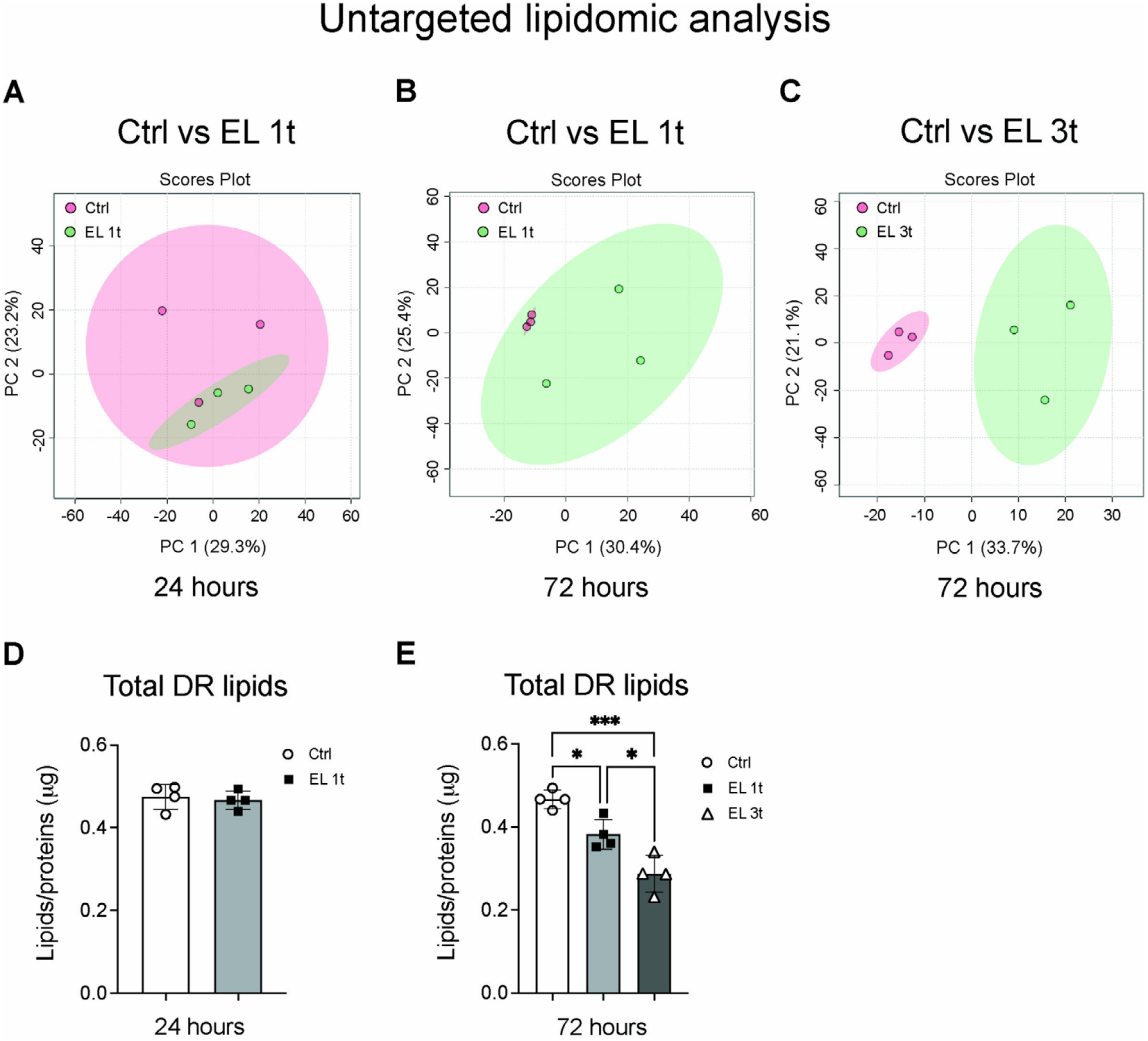
Untargeted lipidomic analysis by LCMS/MS: PCA analysis and differentially regulated lipids. Multivariate analysis of changes in total lipids following electrical stimulation in SH‐SY5Y cells. Principal component analysis (PCA) of control cells compared to electrically stimulated cells at 24 h (A), 72 h (B), and after multiple stimulation at 72 h (C); *n* = 3. Total differentially regulated (DR) lipids in controls compared to single treatment at 24 h (D) and in controls compared to multiple treatment at 72 h (panel E). Data are presented as mean ± SEM, and each point in the graphs represents an experimental replicate. Statistical significance was determined by a two‐tailed Student's *t*‐test or ordinary one‐way ANOVA. *p < 0.05; ***p < 0.001; *n* = 4.

To investigate the effects of electrical stimulation on cellular metabolism and homeostasis, we performed lipidomic analysis by liquid chromatography‐mass spectrometry (LCMS/MS) on electrically stimulated and non‐stimulated cells. Principal component analysis (PCA) showed a partial overlap between the control and EL 1t groups at 24 h after DCS (Figure 2A), with a clearer separation observed at 72 h (Figure 2B) compared to control. The separation between the lipid profiles of stimulated and non‐stimulated cells becomes significantly different when stimulation is repeated, indicating more pronounced lipidomic shifts with multiple stimulations (Figure 2C). Quantification of differentially regulated (DR, *p* < 0.05) lipids showed no significant difference between control and stimulated cells after 24 h (Figure 2D). However, a significant decrease in total DR lipid content was observed after 72 h. In the EL 1t group, a reduction of about 15% was observed compared to the control, while in the EL 3t group, total DR lipids were reduced by about 25% compared to the control. In addition, the EL 3t group showed a further statistically significant decrease in total DR lipids when compared to the EL 1t group (Figure 2E).

**FIGURE 3:**
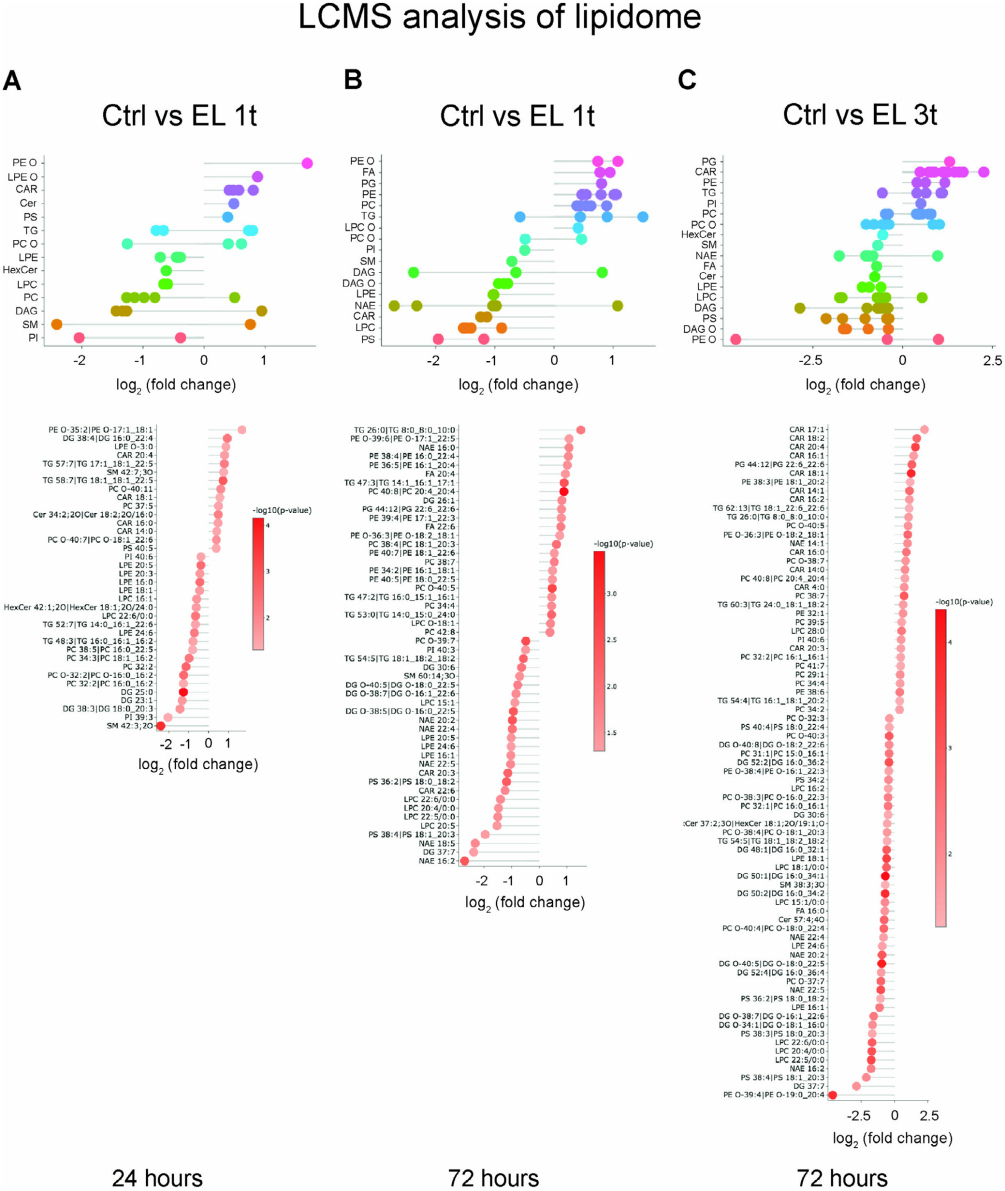
Untargeted lipidomic analysis by LCMS/MS: Differentially regulated lipids by classes. Lollipop diagrams showing modulated lipid species, grouped by classes (upper panels; A: EL 1t, 24 h; B: EL 1t, 72 h; C: EL 3t, 72 h) expressed as Log_2_fold changes (*p* < 0.05). Up‐ and downregulated single species indicated for each class (lower panels; A: EL 1t, 24 h; B: EL 1t, 72 h; C: EL 3t, 72 h) expressed as Log_2_fold changes (*p* < 0.05). The different color of each single point indicates log_10_(*p*); *n* = 4.

Any given lipid species belongs to a specific class, each of which plays a specific role in the cell. To fully understand how DCS alters cellular metabolism, it is important to consider not only the abundance of these lipid species and classes but also their relative contribution to the changes compared to the control group, as well as the number of regulated species within each class. Therefore, we evaluated the log_2_ fold changes (log_2_FC) in lipid concentrations between stimulated and non‐stimulated cells, either 24 or 72 h after a single stimulation or 24 h after multiple stimulations. The lollipop diagrams show the significantly modulated lipid species, grouped by classes, and highlight the most important changes (Figure 3A–C). The DR lipid classes vary across comparisons, indicating that lipid remodeling is time‐dependent. After a single stimulation, all DR lipid classes show changes in the range of −2.5 < log_2_FC < +1 (Figure 3A,B). With repeated stimulation, however, the changes become more pronounced and reach −3 < log_2_FC < +2.5 (Figure 3C). These graphs show both up‐ and downregulated lipid species compared to untreated cells, with the number of DR species indicated for each class (Figure 3A–C; lower panels). 24 h after a single stimulation, 15 species were upregulated and 21 downregulated (Figure 3A), while after 72 h 24 species were upregulated and 26 downregulated (Figure 3B). Conversely, after multiple stimulations, 33 lipid species were upregulated and 44 downregulated compared to the control (Figure 3C). Thus, the response to stimulation: (i) leads to downregulation of a larger number of species compared to the upregulated ones; (ii) the number of DR species increases with the duration of stimulation; (iii) this number increases further when stimulation is repeated.

**FIGURE 4:**
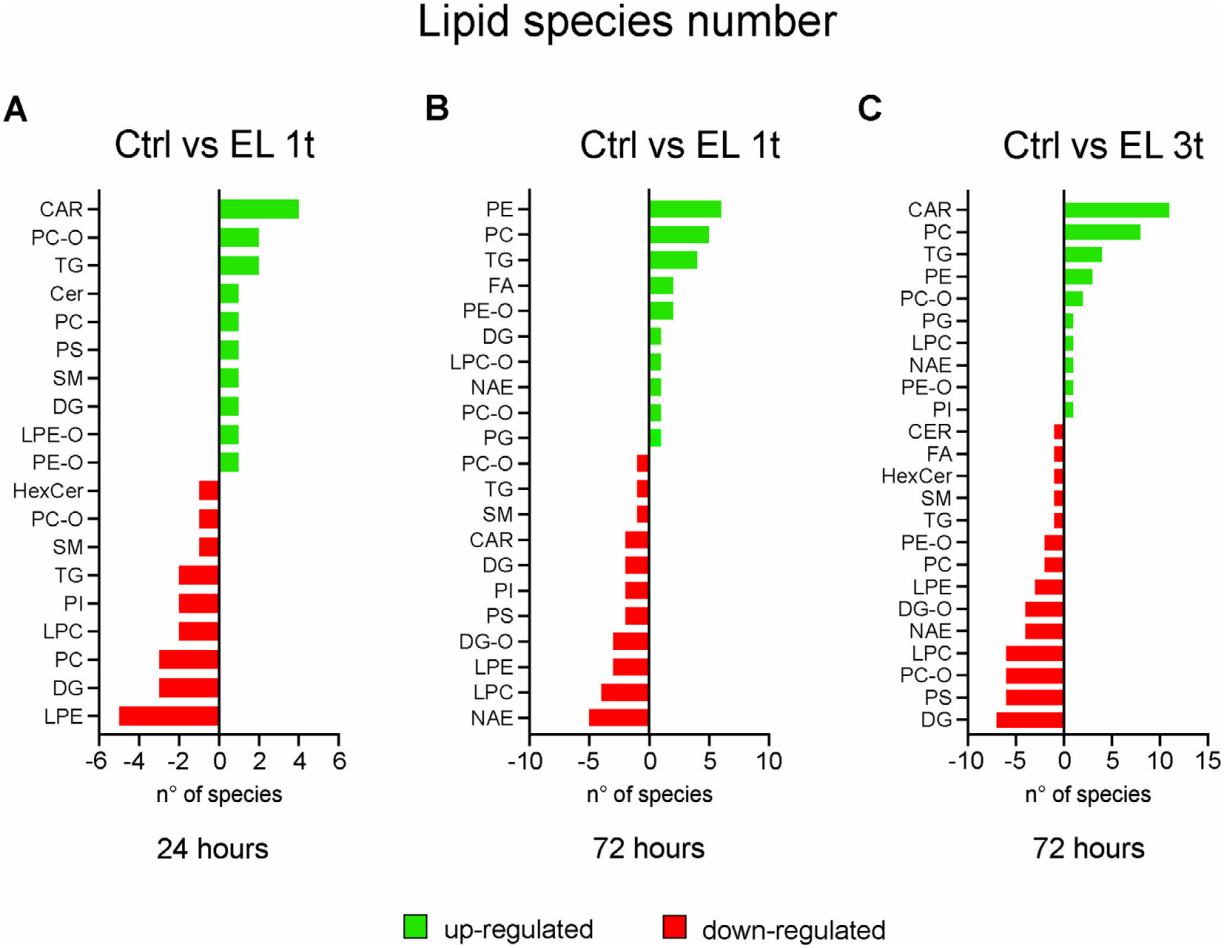
Untargeted lipidomic analysis by LCMS/MS: Number of differentially regulated lipids by classes. Bar graphs showing the number of differentially regulated single species in DCS‐treated cells versus control. Upregulated species: Red bars; downregulated species: Green bars. (A) EL 1t, 24 h; (B) EL 1t, 72 h; (C) EL 3t, 72 h; *n* = 4.

Further analysis revealed a modulation of anionic lipids. With a single stimulation, phosphatidylserine (PS) was upregulated after 24 h and downregulated after 72 h, while phosphatidylinositol (PI) decreased at both time points (Figure 4A,B). Phosphatidylethanolamine (PE), a particular membrane lipid that is known for its cone‐shaped form, increased after 72 h, while its deacylated form, lysophosphatidylethanolamine (LPE), was reduced (Figure 4B). Similarly, phosphatidylcholine (PC), a major membrane lipid, after a transient decrease at 24 h, increased at 72 h, while its deacylated form, lysophosphatidylcholine (LPC), decreased. Ether‐linked phosphatidylethanolamine (PE‐O) was upregulated at both time points, while ether‐linked phosphatidylcholine (PC‐O) showed mixed regulation. Sphingomyelin (SM), another major building block among membrane lipids, was initially modulated but decreased after 72 h. After 24 h, ceramide transiently increased, while monoglycosylated ceramide (HexCer) decreased. Triglyceride (TG) and diglyceride (DG) showed an initial modulation, followed by an overall increase in TG after 72 h, alongside a decrease in acylcarnitine (CAR), an increase in fatty acid (FA), and a decrease in *N*‐acylethanolamine (NAE).

When the cells underwent multiple stimulations, the response of the anionic lipids was different. PS was downregulated, while PI was upregulated (Figure 4C). Within the PC‐O and PE‐O classes, the majority of species were downregulated. SM, ceramide (Cer), and HexCer were also down‐regulated. In contrast, most triglyceride (TG) species were upregulated, while DAG and ether‐linked diglyceride (DAG‐O) were reduced. This reduction in DAG was accompanied by an increase in CAR and a decrease in FA. Most significantly, NAE species were predominantly downregulated (Figure 4C).

**FIGURE 5:**
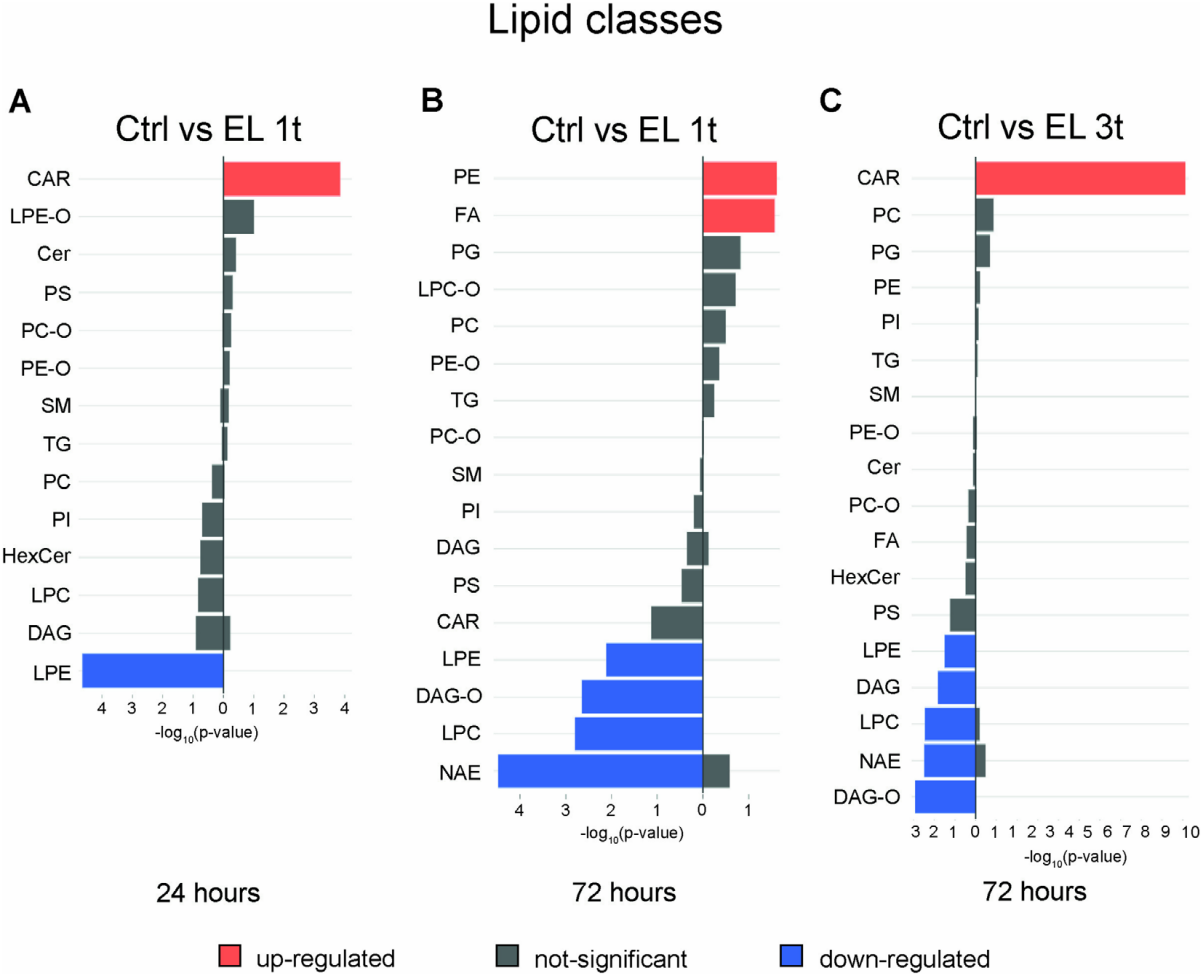
Fisher exact test of lipid class changes in stimulated versus unstimulated cells. Bar graphs showing differentially regulated species (−log_10_ *p*‐value) in DCS‐treated cells versus control. Upregulated species: Orange bars; downregulated species: Blue bars. Gray bars represent not relevant changes according to the Fisher test. (A) EL 1t, 24 h; (B) EL 1t, 72 h; (C) EL 3t, 72 h; *n* = 4.

The overall change of a lipid class is influenced by the combined contributions of all species within that class, which may differ in their degree of up‐ or downregulation, and by the statistical significance of these changes. To assess the significance of the DR lipid classes, we performed a Fisher test. The more significant changes in the upregulated (*orange bars*) and downregulated (*blue bars*) species are shown in Figure 5. After a single stimulation, CAR is significantly upregulated, while LPE is significantly downregulated within 24 h (Figure 5A). However, 72 h after stimulation, CAR has an opposite modulation, although not significant. Other species, including LPE, LPC, ether‐linked DAG‐O, and NAE, show marked downregulation, while PE and FA are strongly upregulated (Figure 5B). Upon multiple stimulations, upregulation of the CAR class again becomes significant, while LPE, LPC, DAG, DAG‐O, and NAE show marked downregulation (Figure 5C). Thus, the transient upregulation of CAR and LPE observed after a single stimulus at 24 h persists after repeated stimulation. These changes are associated with a marked downregulation of deacylated glycerolipids and glycerophospholipids and a reduction in NAE.

Figures 3, 4, and 5 show that DCS causes lipid modulation that develops over a 72‐h period, with the number of affected species increasing over time and with repeated stimulation. In particular, the pro‐inflammatory lipids LPE and LPC are downregulated after both single and repeated stimulations. However, while the downregulation of LPE is statistically significant in both cases (based on the Fisher test), LPC only reaches statistical significance after multiple stimulations (Figure 5B,C). In addition, the pro‐inflammatory DR ceramide containing palmitate, a fatty acid commonly produced by de novo synthesis, shows an increase 24 h after a single stimulation but not after multiple stimulations. Furthermore, significant upregulation of 11 CAR species occurs with a significant decrease in diacylglycerols (both DG and DAG‐O), along with a decrease in the FA palmitate after multiple stimulations, suggesting that lipid mobilization and oxidation rather than inflammation‐induced synthesis is occurring. Furthermore, 8 of the 10 DR species of PC are upregulated by multiple stimulations, while 6 of the 8 PC‐O species are downregulated. Similarly, 3 DR species of PE are upregulated, while 2 of 3 PE‐O species are downregulated, suggesting membrane remodeling aimed at increasing the accumulation of ester bonds. This remodeling probably improves membrane plasticity, allowing the cells to respond better to external stimuli. In this context, the modulation of the anionic lipids PS and PI shows opposite trends 24 h after single and multiple stimulations, respectively.

**FIGURE 6:**
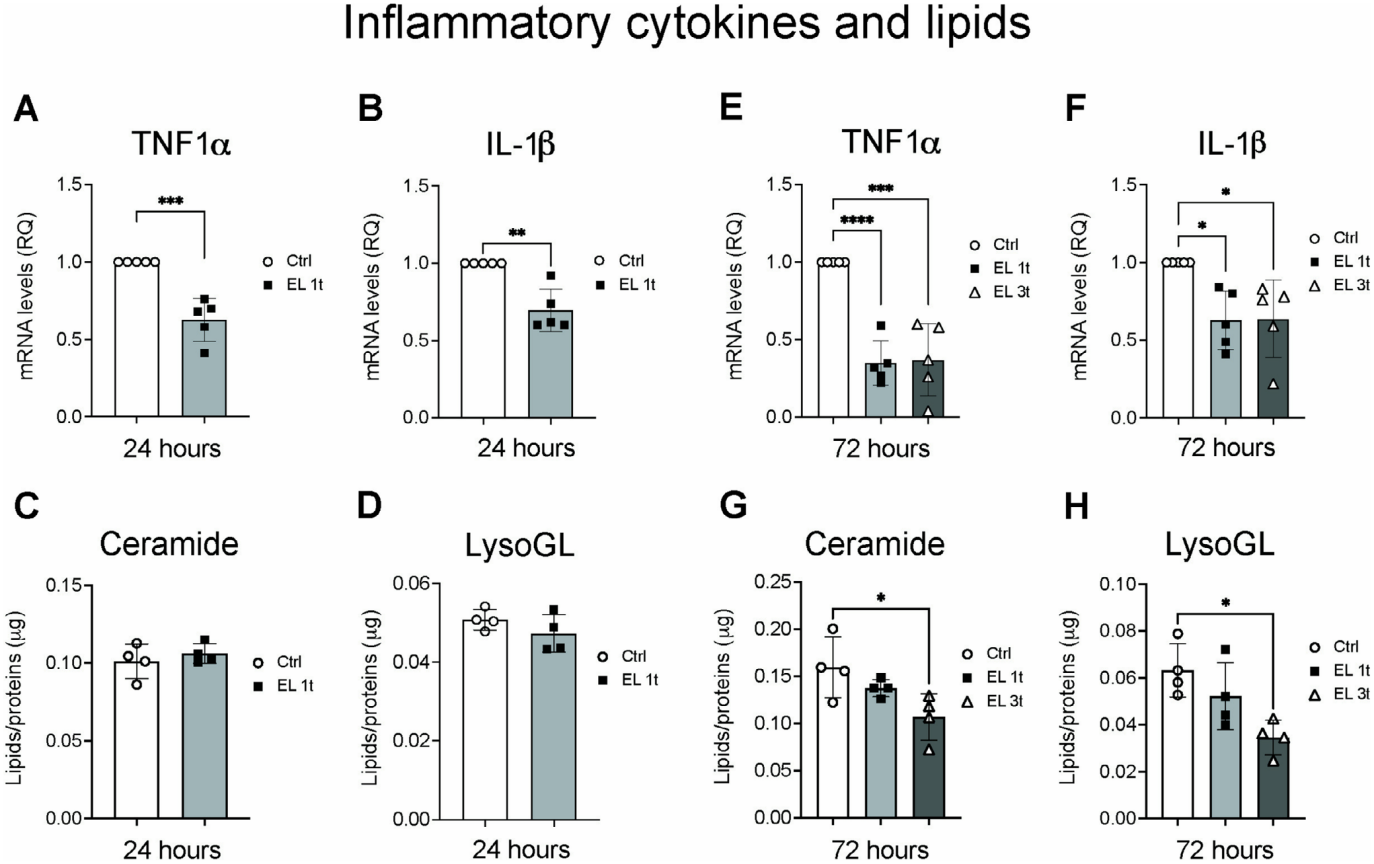
DCS effects on inflammation. Gene expression analysis of inflammatory cytokines TNF‐α and IL‐1β mRNA after 24 h (A and B) or 72 h after single or multiple DCS stimulation (E and F) measured by Real‐Time PCR. Values were normalized on GAPDH mRNA expression. Data are expressed as relative quantities compared with control cells not exposed to DSC treatment. Relative amounts of inflammatory lipid ceramides and lyso‐PC by untargeted LCMS, after 24 h (C and D) and 72 h after single or multiple DCS stimulation (G and H). Data are presented as mean ± SEM, and each point in the graphs represents an experimental replicate. Statistical significance was determined by a two‐tailed Student's t‐test or ordinary one‐way ANOVA. *p < 0.05; **p < 0.01; ***p < 0.001; ****p < 0.0001; *n* = 4.

To prove this hypothesis, we first assessed the expression of two major inflammatory cytokines, tumor necrosis factor‐α (TNF‐α) and interleukin‐1β (IL‐1β), and compared them with the relative amount of two classes of inflammatory lipids (Ceramides and lysoglycerol lipids). The results showed a significant reduction in mRNA levels of TNF‐α and IL‐1β 24 h after a single stimulation (EL 1t) compared to controls (Ctrl) (Figure 6A,B). As for lipid modulation at the 24‐h time point, both ceramide and lysoglycerol lipid (lysoGL) levels remained unchanged (Figure 6C,D). After 72 h, the expression of TNF‐α remains significantly reduced after both single (EL 1t) and multiple stimulations (EL 3t) (Figure 6E). IL‐1β expression is also significantly reduced after both stimulation treatments (Figure 6F). Lipid analysis after 72 h shows a decrease in both ceramide and lysoGL compared to the controls, with multiple stimulations showing a statistically significant effect (Figure 6G,H).

**FIGURE 7:**
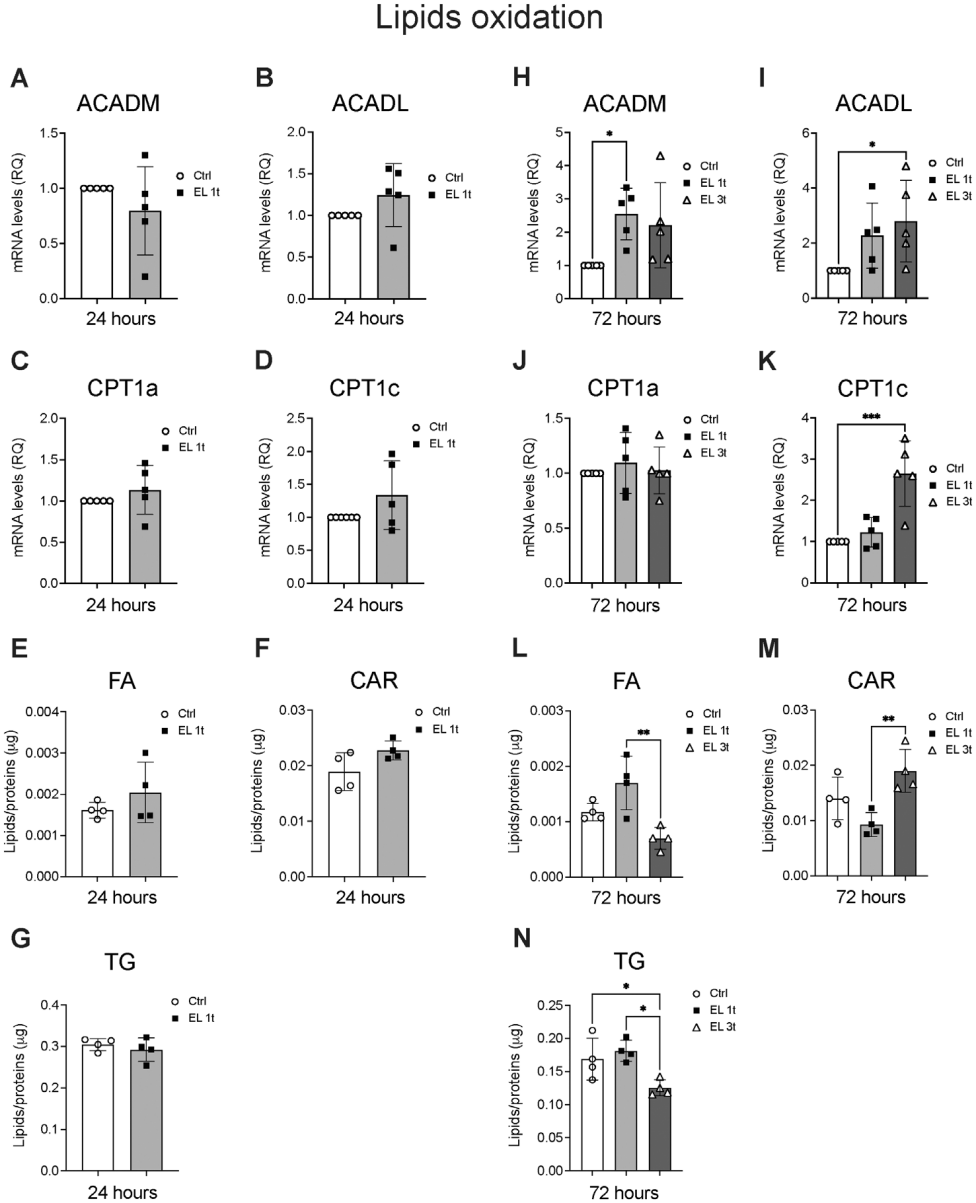
DCS effects on mitochondrial activities. Gene expression analysis of FA Acyl‐CoA dehydrogenases for medium‐ and long‐chain FA (ACADM and ACADL) and Carnitine Palmitoyl transferase 1a and 1c (CPT1a and CPT1c) after 24 h (A–D) or 72 h after single or multiple DCS stimulation (H–K) measured by Real‐Time PCR. Values were normalized on GAPDH mRNA expression; *n* = 5. Data are expressed as relative quantities compared with control cells not exposed to DSC treatment. Relative amounts of FA, CAR, and TG were measured by untargeted LCMS after 24 h (E–G) or 72 h after single or multiple DCS stimulation (L–N); *n* = 4. Data are presented as mean ± SEM, and each point in the graphs represents an experimental replicate. Statistical significance was determined by a two‐tailed Student's *t*‐test or ordinary one‐way ANOVA. *p < 0.05; **p < 0.01; ***p < 0.001.

To demonstrate the role of mitochondrial activities in response to DCS stimulation, we measured the effects on the expression of lipid oxidation‐related genes and lipid species at both 24 and 72 h. At 24 h after a single stimulation (EL 1t), there were no significant changes in the expression of carnitine palmitoyl transferase 1a and 1c (CPT1a and CPT1c) and acyl‐CoA dehydrogenases (ACADs) (for medium, ACADM, and long, ACADL, chains FA) between stimulated cells and controls (Figure 7A–D). Lipid analysis after 24 h also shows no significant changes in FA, CAR, or TG levels (Figure 7E–G). After 72 h, the expression of lipid oxidation genes showed more significant changes. ACADM and ACADL were upregulated after both single and multiple stimulations. However, the expression of ACADM was significantly increased after a single stimulation (Figure 7H), while the upregulation of ACADL expression appeared to be promoted by multiple stimulations (Figure 7I). In addition, CPT1c was strongly upregulated after multiple stimulations (Figure 7K), while CPT1a remained unchanged (Figure 7J). Lipid analysis after 72 h showed a significant decrease in FA and TG levels, especially after multiple stimulations (Figure 7L,N), while CAR levels only increased significantly when cells were stimulated with multiple stimulations (Figure 7M). Overall, these results suggest that DCS promotes lipid oxidation by upregulating key genes involved in fatty acid metabolism and modulating lipid levels over time.

**FIGURE 8:**
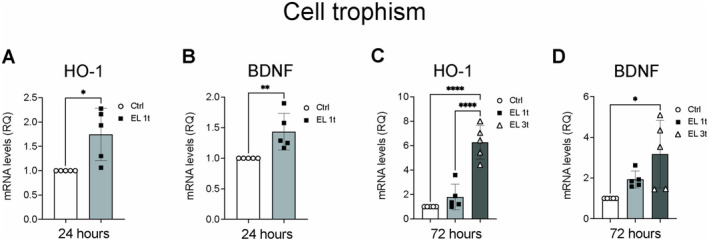
DCS effects on cell trophism. Gene expression analysis of heme oxygenase (HMOX/HO‐1) and brain‐derived neurotrophic factor (BDNF) after 24 h (A, B) or 72 h after single or multiple DCS stimulation (C, D) measured by Real‐Time PCR. Values were normalized on GAPDH mRNA expression. Data are expressed as relative quantities compared with control cells not exposed to DSC treatment. Data are presented as mean ± SEM, and each point in the graphs represents an experimental replicate. Statistical significance was determined by a two‐tailed Student's *t*‐test or ordinary one‐way ANOVA. *p < 0.05; **p < 0.01; ****p < 0.0001; *n* = 5.

Next, we examined the response to stimulation of key proteins that regulate cell trophism, such as heme oxygenase (HMOX/HO‐1), an enzyme involved in oxidative stress, and brain‐derived neurotrophic factor (BDNF), an important neuronal survival factor. After 24 h, cells exposed to a single treatment with EL (EL 1t) showed a significant increase in HMOX/HO‐1 expression (Figure 8A) and an upregulation of BDNF levels (Figure 8B) compared to the control group. After 72 h, an even more pronounced effect was observed, with both a single (EL 1t) and multiple (EL 3t) treatments significantly increasing HMOX/HO‐1 mRNA expression (Figure 8C). Conversely, BDNF levels were upregulated but only reached statistical significance with repeated stimulation (EL 3t) (Figure 8D). These results indicate that EL had a time‐ and dose‐dependent effect on trophic factor expression, suggesting a potential neuroprotective and growth‐promoting role of EL in cells over time.

**FIGURE 9:**
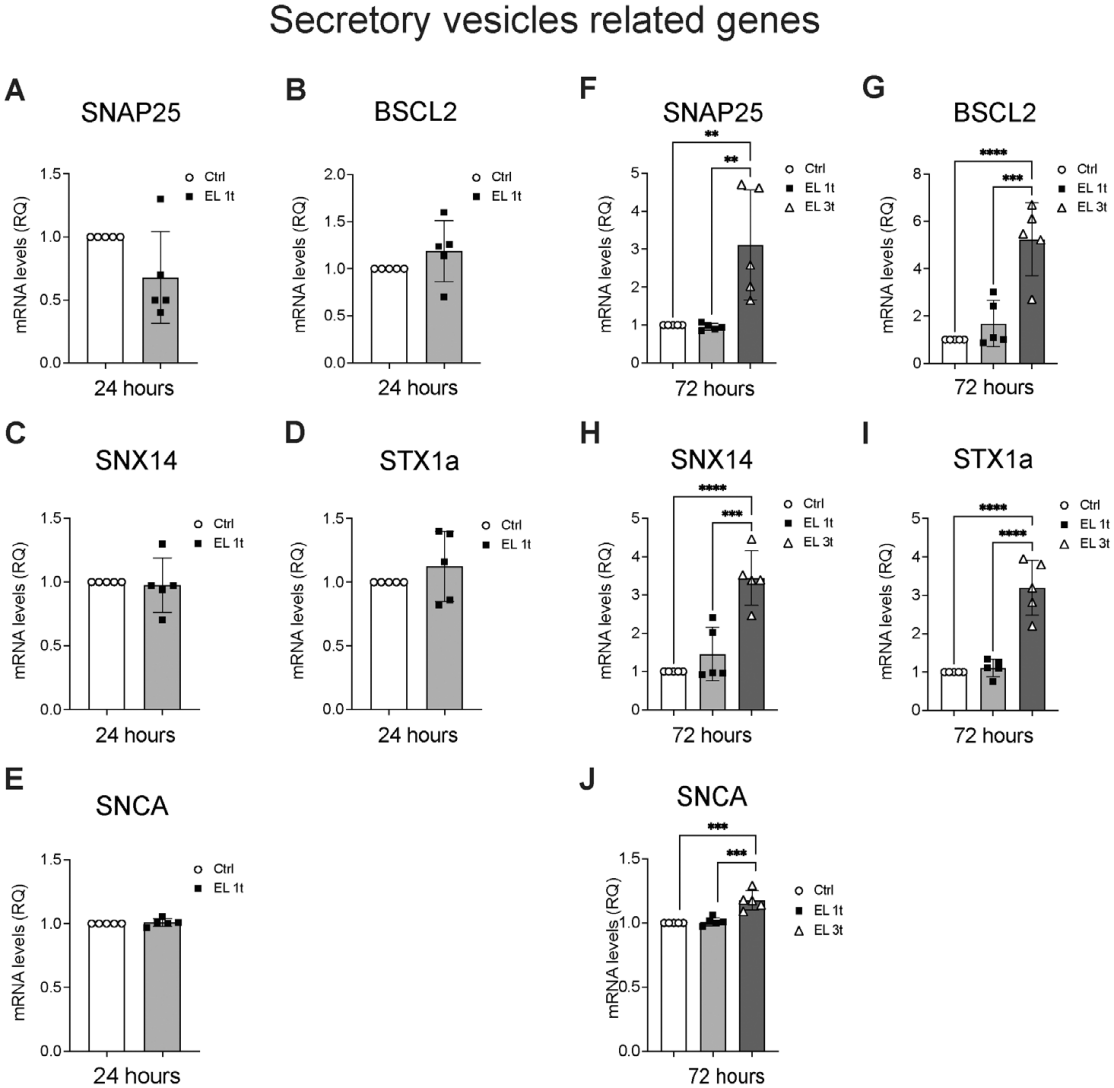
DCS effects on vesicular biogenesis and transport. Gene expression analysis of the SNARES genes synaptosome‐associated protein (SNAP25), lipid droplet biogenesis‐associated (BSCL2), sorting nexin 14 (SNX14), syntaxin 1A (STX1A), and α‐synuclein (SNCA) after 24 h (A–E) or 72 h after single or multiple DCS stimulation (F–J) was measured by Real‐Time PCR. Values were normalized on GAPDH mRNA expression. Data are expressed as relative quantities compared with control cells not exposed to DSC treatment. Data are presented as mean ± SEM, and each point in the graphs represents an experimental replicate. Statistical significance was determined by a two‐tailed Student's *t*‐test or ordinary one‐way ANOVA. **p < 0.01; ***p < 0.01; ****p < 0.0001; *n* = 5.

Finally, the functional role of these metabolic and phenotypic changes was analyzed by examining the expression of SNAP receptor proteins (SNARE), which are involved in vesicle transport and synaptic transmission and are known to be downregulated in undifferentiated and stressed neurons (Madrigal et al. 2019). After 24 h, the single EL treatment (EL 1t) does not significantly alter the expression of synaptosome‐associated protein (SNAP25), lipid droplet biogenesis‐associated (BSCL2), sorting nexin 14 (SNX14), syntaxin 1A (STX1A), and of the SNARE proteins complex stabilizer α‐synuclein (SNCA), compared to the control group (Figure 9A–E). However, after 72 h, there was a significant increase in the expression of these genes in response to multiple EL treatments (EL 3t) compared to the Ctrl and EL 1t groups (Figure 9F–J).

**FIGURE 10:**
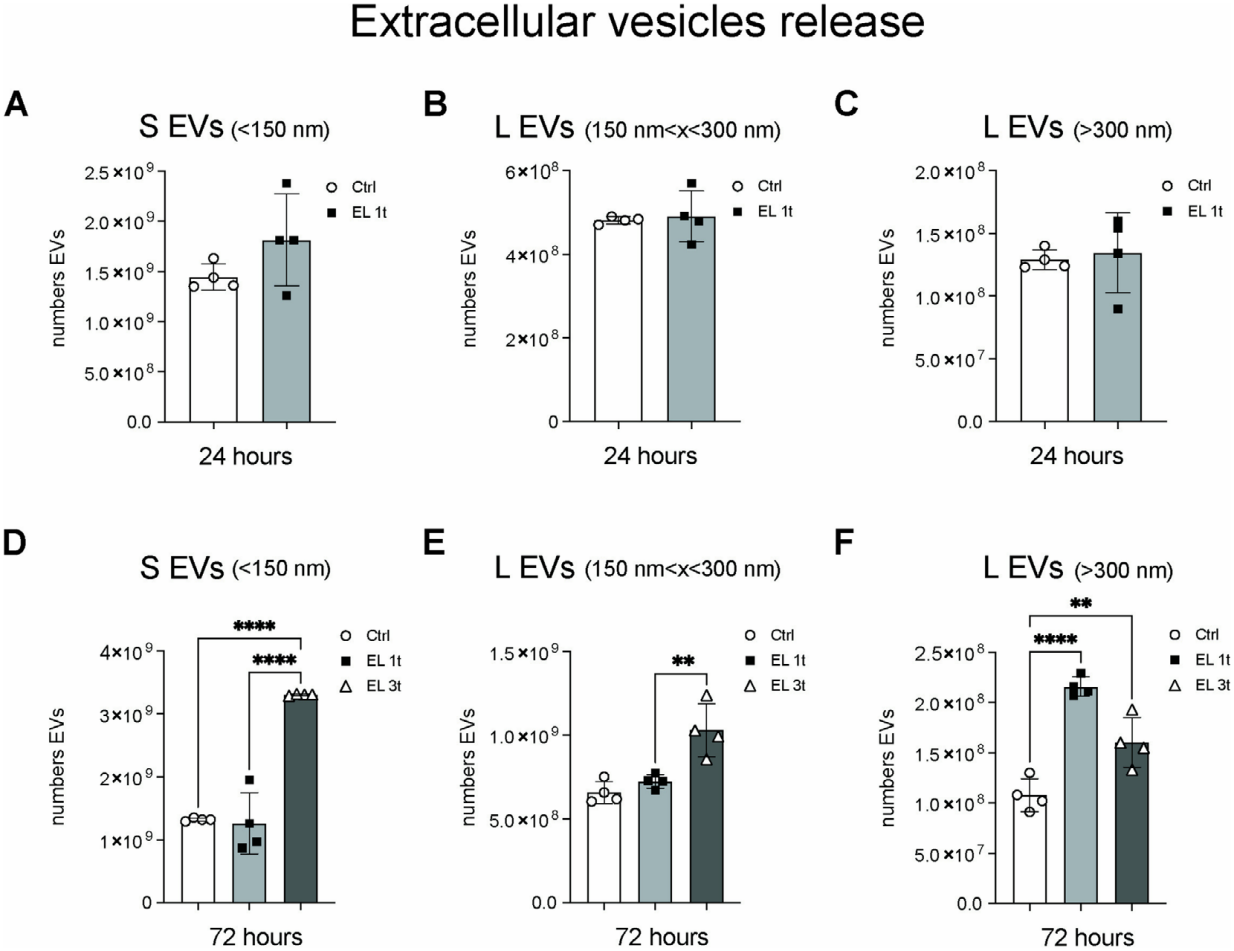
DCS effects on vesicular release. Nanoparticle tracking analysis (NTA). Concentration of vesicles released in the culture medium by a single DCS stimulation medium after 24 h: (A) small vesicles (diameter below 150 nm); (B) large vesicles (diameter between 150 and 300 nm); (C) large vesicles (diameter above 300 nm). Concentration of vesicles released in the culture medium by a single or multiple DCS stimulation after 72 h: (D) small vesicles (diameter below 150 nm); (E) large vesicles (diameter between 150 and 300 nm); (F) large vesicles (diameter above 300 nm). Data are presented as mean ± SEM, and each point in the graphs represents an experimental replicate. Statistical significance was determined by a two‐tailed Student's t‐test or ordinary one‐way ANOVA. **p < 0.01; ****p < 0.0001; *n* = 4.

In addition, the release of extracellular vesicles (EVs) was investigated. EVs were isolated by an ultracentrifugation protocol and characterized by nanoparticle tracking analysis (NTA) and marker enrichment (Figure S1). While a single stimulus did not trigger a significant release of EVs after 24 h (Figure 10A–C), a more pronounced effect was observed after 72 h. The release of large EVs (L EVs), with a diameter larger than 300 nm, was significantly increased in both the single treatment group (EL 1t) and the multiple treatment group (EL 3t) compared to the controls (Figure 10F). Multiple stimulations led to increased secretion of both small EV (S EVs) with a diameter of less than 120 nm (exosomes) and large vesicles with an intermediate diameter between 120 and 300 nm (Figure 10D,E).

## 4 Discussion

This study investigated the effects of direct current stimulation (DCS) on cellular metabolism and signaling pathways. SH‐SY5Y neuroblastoma cells were exposed to weak direct current for 20 min, and changes were observed at both short (24 h) and long (72 h) time points. To simulate the therapeutic application of DCS, where multiple cycles are common, cells were also stimulated three times 24 h apart, with analysis 24 h after the last stimulation, to compare single and multiple treatments. The results showed significant lipid modulation after both single and multiple DCS and revealed time‐ and dose‐dependent effects on lipid profiles that could have implications for cellular homeostasis, neuroplasticity, and inflammation.

Significant lipidomic changes were observed by untargeted mass spectrometry analysis, depending on the timing and frequency of DCS. After a single stimulation, lipid remodeling appeared relatively mild, with a partial separation of lipid profiles between stimulated and control groups after 24 h, which became more pronounced after 72 h. In addition, lipid species affected by DCS also differed between the 24‐ and 72‐h time points, with an overall reduction in total lipid mass observed at 72 h. In particular, a single stimulation at 72 h reduced the amounts of anionic lipids such as phosphatidylserine (PS) and phosphatidylinositol (PI), possibly impacting membrane remodeling. Phosphatidylethanolamine (PE), a cone‐shaped zwitterionic lipid that is enriched in the inner leaflet of membranes and causes negative curvature, was increased after both single and multiple stimulations (Males et al. 2024). Interestingly, PE is crucial for protein folding, mitochondrial respiratory chain function, and membrane autophagy, and its level is known to decrease with age and in neurodegenerative diseases such as Parkinson's (Riekkinen et al. 1975; Wang et al. 2014).

In addition, ether‐bound glycerophospholipids, such as PE‐O, PC‐O, and LPC‐O, were also increased after stimulation. The increased presence of ether‐bound fatty acids could provide greater membrane stability and thus inhibit the activity of esterases, as shown by the decreased amounts of deacylated lipids such as LPC and LPE. After 24 h, ceramide, especially C16 ceramide, was increased, possibly due to sphingolipid synthesis (Mingione et al. 2021). This accumulation suggests the induction of an initial inflammatory response, while the remodeling of triglycerides (TG), diacylglycerol (DG), and the increase in acylcarnitine (CAR) at 24 h may reflect increased lipid oxidation in the mitochondria, which attenuates at later time points.

With repeated stimulation, the overall lipid reduction was more pronounced, and the modulation of anionic lipids differed compared to single stimulation. PE increased again, accompanied by a significant increase in 11 CAR species, a decrease in DG, and a lower free palmitate content. This suggests that lipid mobilization and oxidation persist after 72 h, opposing lipid synthesis. Remarkably, C16 ceramide was no longer upregulated, and instead one ceramide and one glycosylated ceramide species were downregulated. Furthermore, multiple stimulations downregulated most PC‐O and PE‐O species, while PC and PE were upregulated, indicating membrane remodeling that favors the accumulation of ester bond‐bearing lipids. This remodeling likely enhances membrane plasticity, as ether bonds are more resistant to degradation, while ester bonds confer greater fluidity and adaptability. These properties are essential for membrane functions such as vesicular trafficking, signal transduction, and dynamic processes like the release of extracellular vesicles (EVs) (Guler et al. 2009; Dean and Lodhi 2018; Papin et al. 2023).

The lipidomic changes correlated with transcriptional regulation of genes involved in inflammation and energy production. Both TNF‐α and IL‐1β expression were significantly reduced, along with a decrease in ceramides and lyso‐glycerophospholipids, which are involved in inflammation‐related signaling (Khan and Ilies 2023; Quinville et al. 2021) and neuromodulation (Pan et al. 2023). A single stimulation led to a transient increase in the expression of stress response genes such as HO‐1 and the neurotrophic factor BDNF. These increases persisted after 72 h following multiple stimulations, suggesting that repeated treatment may promote antioxidant activities, neuroprotection, and neuronal plasticity. Along with these results, an increase in the transcription levels of genes associated with lipid oxidation was also observed. In this context, lipid oxidation and energy gain counteract lipid accumulation and peroxidation, which significantly contribute to neurodegenerative diseases (Angelova, Esteras, and Abramov 2021).

Multiple stimulations led to an upregulation of CPT1c and ACADL, in parallel with increased CAR levels and reduced fatty acids (FA) and TG. CPT1c, a brain‐specific fatty acid transporter involved in brain energy homeostasis, synaptic plasticity, and stress response, was strongly induced, as was ACADL, which facilitates fatty acid oxidation, a key process in energy metabolism (Price et al. 2002; Fado et al. 2023; Iborra‐Lazaro et al. 2023; Narayanan et al. 2024).

DCS thus triggered transient inflammation and remodeling of the membrane lipids, in particular increasing the ratio of ether to ester bonds. Multiple stimulations reversed this ratio and promoted membrane curvature through increased PE, enhanced mitochondrial lipid import, and oxidation. Inflammatory lipid and cytokine transcription was reduced, while BDNF transcription increased, indicating a possible neuroprotective effect.

Overall, these results are consistent with previous studies from our group that have shown that DCS has a homeostatic effect in human neuroblastoma cells by modulating macroautophagy and chaperone‐mediated autophagy on the one hand and reducing polymeric α‐synuclein on the other (Sala et al. 2021). Furthermore, our results are in line with recent studies on the homeostatic effects of DCS on β‐amyloid protein (Yu et al. 2015; Luo et al. 2020) and the neurovascular unit (Luo et al. 2022).

DCS therapy therefore exerts its effect primarily through neuromodulation, favoring the recovery of neuronal networking. Recently, in addition to the release of neurotransmitters mediated by synaptic activity, EVs have also emerged as key players in interneuronal communication. Neuron‐derived EVs can interact specifically with other neurons, representing a novel mechanism of neuronal signaling (Chivet et al. 2014). Both extracellular vesicles and neurotransmitter vesicles rely on the SNARE protein system for docking and release (Liu et al. 2023; Caruel and Pincet 2024). The SNARE complex consists of proteins anchored in the plasma membrane, such as syntaxin‐1 (STX1a and 1b) and SNAP‐25, as well as the vesicle‐associated membrane protein synaptobrevin‐2 (VAMP/SYB2). Alpha synuclein, encoded by the SNCA gene, is a presynaptic protein that binds and stabilizes the SNARE complex, exerting an essential role in modulating the formation and dimensions of presynaptic vesicles (Burre et al. 2010). Deficiencies in α‐synuclein, SNARE proteins, and related factors are associated with various neurological diseases, including Alzheimer's and Parkinson's disease (Shu et al. 2024; Meloni et al. 2023). SNAP25 plays a crucial role in the fusion of synaptic vesicles and multilamellar bodies with the membrane, facilitating the release of neurotransmitters and enhancing EV‐mediated intercellular communication (Liu et al. 2023; Agliardi et al. 2019). SNX14, a member of the sorting nexin family, is involved in vesicular trafficking and is highly expressed in the nervous system, contributing to both excitatory and inhibitory synaptic transmission (Carlton et al. 2005; Huang et al. 2014). Seipin/BSCL2, an endoplasmic reticulum (ER) protein, is expressed in various tissues, including the central nervous system and adipose tissue (Zhou et al. 2020). Loss of seipin function leads to lipodystrophy type II (Berardinelli‐Seip syndrome), and mutations in the gene are associated with a variety of motor neuropathies (Agarwal and Garg 2006; Auer‐Grumbach et al. 2005; Guo et al. 2013). Seipin also regulates the formation of lipid droplets, sphingolipids, and myelin, influences synaptic transmission, and possibly promotes the response to oxidative stress (Sanchez‐Iglesias et al. 2019; Cui et al. 2024; Amarasinghe et al. 2023). In this context, the increased expression of SNCA, STX1a, SNAP25, SNX14, and BSCL2 after multiple DCS stimulations suggests that the transcriptional regulation induced by repeated treatment may promote vesicle trafficking and possibly intercellular communication. To confirm this hypothesis, we examined the release of EVs in the medium of stimulated cells and identified three populations of EVs based on their diameter. While larger EVs (> 300 nm) increased after 72 h with both single and multiple treatments, we observed an increased release of small EVs (< 120 nm diameter) after multiple stimulations, suggesting that changes in lipid metabolism, energy supply, and transcriptional regulation could mediate the neuroprotective effect of DCS.

**SCHEME 1:**
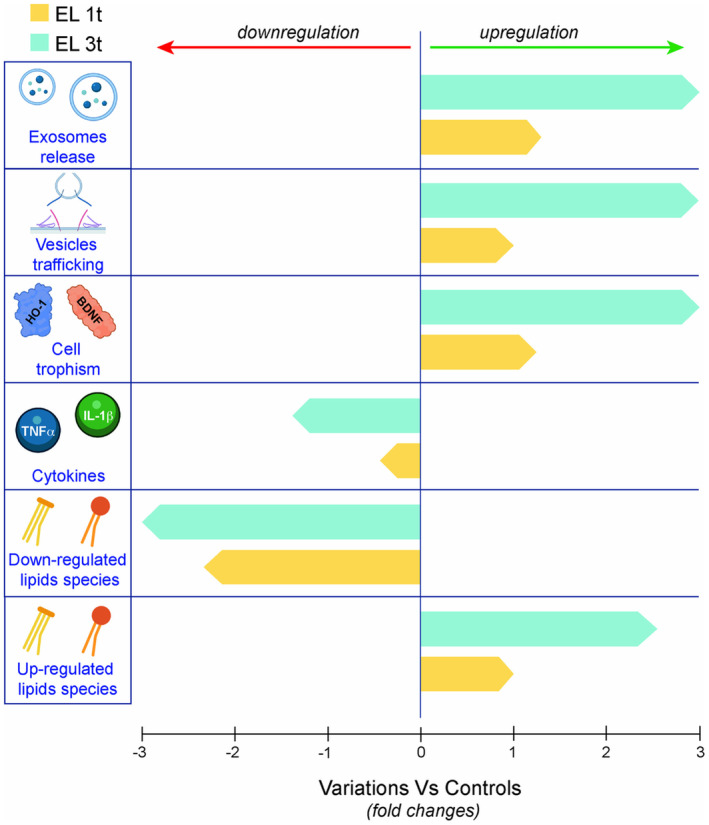
Major findings: Range of fold changes in the variation of differentially regulated lipid species, inflammation‐related cytokines (TNF‐α and IL‐1β transcription), cell trophism factors (BDNF and HMXO/HO1 transcription), vesicle trafficking and biogenesis (SNARE transcription), and exosome release in SHSY‐5Y cells after single (EL 1t) or multiple (EL 3t) DCS as compared to control.

In summary, DCS induces transient changes in lipid metabolism with minimal phenotypic effects after a single stimulation. However, repeated stimulations lead to more extensive and profound lipid modulation, transcriptional regulation of genes associated with inflammation and neuroprotection, increased vesicle formation, and increased exosome release (Scheme 1). These results suggest that DCS cycles can train neuronal cells to adopt a neuroprotective and neuroplastic‐orientated phenotype by modulating structural and metabolic pathways. At the cellular and molecular level, this could support the therapeutic effects of tDCS in patients with neurodegenerative diseases, as recent studies have translated these findings to human models (Dhaynaut et al. 2022), suggesting a broad disease‐modifying and neuroprotective effects of direct current polarization.

## Author Contributions

**Marco Piccoli:** writing – review and editing, writing – original draft, data curation, supervision, investigation, methodology. **Luisa Barbato:** investigation, writing – original draft, methodology, writing – review and editing, data curation, supervision. **Natale Vincenzo Maiorana:** supervision, conceptualization. **Alessandra Mingione:** investigation, data curation, methodology, supervision. **Francesca Raimondo:** investigation, formal analysis. **Marco Ghirimoldi:** conceptualization, formal analysis, data curation. **Federica Cirillo:** conceptualization, supervision, writing – review and editing. **Mattia Schiepati:** investigation, formal analysis. **Domenico Salerno:** investigation, formal analysis. **Luigi Anastasia:** supervision, writing – review and editing. **Elisabetta Albi:** writing – review and editing, supervision, conceptualization. **Marcello Manfredi:** investigation, conceptualization, methodology, formal analysis, supervision, data curation, writing – review and editing. **Tommaso Bocci:** conceptualization, supervision. **Alberto Priori:** conceptualization, supervision, writing – review and editing, funding acquisition. **Paola Signorelli:** conceptualization, funding acquisition, writing – original draft, writing – review and editing, supervision, data curation, project administration.

## Conflicts of Interest

The authors declare no conflicts of interest.

## Supporting information


Data S1.

## Data Availability

Datasets related to the present study are available upon reasonable request from interested researchers.
