# Supplementary material for: Direct Current Stimulation (DCS) Modulates Lipid Metabolism and Intercellular Vesicular Trafficking in SHSY‐5Y Cell Line: Implications for Parkinson's Disease
Source: J Neurochem. 2025 Feb 10;169(2):e70014. doi: 10.1111/jnc.70014 (PMC11811683; doi:10.1111/jnc.70014)
Supplement: Supplementary file 1 — Data S1. [file JNC-169-0-s001.pdf]

Manuscript: JNC-2024-0590

TITLE

Direct Current Stimulation (DCS) modulates lipid metabolism and intercellular vesicular trafficking in SHSY-5Y cell line: implications for Parkinson's disease

Marco Piccoli[1,7#], Luisa Barbato[2#], Natale Vincenzo Maiorana[3], Alessandra Mingione[3], Francesca Raimondo[4], Marco Ghirimoldi[5], Federica Cirillo[1], Mattia Schiepati[3], Domenico Salerno[6], Luigi Anastasia[1,7], Elisabetta Albi[8], Marcello Manfredi[1,5,9], Tommaso Bocci[3], Alberto Priori[3], Paola Signorelli[2,3*].

[1]Institute for Molecular and Translational Cardiology (IMTC), IRCCS Policlinico San Donato, Milan, Italy

[2]Biochemistry Laboratory, IRCCS Policlinico San Donato, Piazza Malan 2, Milan, Italy

[3]"Aldo Ravelli" Research Centre Department of Health Sciences, University of Milan, Italy

[4]School of Medicine and Surgery, University of Milan-Bicocca, Monza, Italy

[5]Biological Mass Spectrometry Lab, Department of Translational Medicine, University of Piemonte Orientale, Novara, Italy

[6]School of Medicine and Surgery BioNanoMedicine Center NANOMIB, University of Milan-Bicocca, Monza, Italy

[7]School of Medicine, University Vita-Salute San Raffaele, Milan, Italy

[8]Department of Pharmaceutical Sciences, Interno Orto Botanico, University of Perugia, Perugia, Italy

[9]Center for Translational Research Autoimmune Diseases and Allergic Diseases, University of Piemonte Orientale, Novara, Italy

[#] These authors contributed equally to this work

[*] Correspondence: paola.signorelli@unimi.it;

Supplementary Table 1

**List of primers**
ACADL
[Forward] *5' – CGCTGTAAAATCCTATTGCTGACA-3'*
[Reverse] *5'- CTGGTCGCGAGGGAATACG-3'*
ACADM
[Forward] *5' -GGGTTCGGGCGATGCT-3'*
[Reverse] *5' -CTGTGATCTCCAATGAAAACGAGA-3'*
BDNF
[Forward] *5' -CTACGAGACCAAGTGCAATCC-3'*
[Reverse] *5' -AATCGCCAGCCAATTCTCTT-3'*
BSCL2
[Forward]*5' -ATGGTCAACGACCCTCCAGTA-3'*
[Reverse] *5' -GCTGACTGTCGGCATATAGGAA-3'*
CPT1A
[Forward]*5'-TGTCCAGCCAGACGAAGAAC-3'*
[Reverse] *5' -CACTTTCAGGGAGTAGCGCA-3'*
CPT1C
[Forward]*5'-GGACTGATGGAGAAGATCAAAGA-3'*
[Reverse] *5'-CACAAACACGAGGCAAACAG-3'*
HO1
[Forward]*5'-TTCAAGCAGCTCTACCGCTC-3'*
[Reverse] *5' -AACGCAGTCTTGGCCTCTTC-3'*
IL1β

[Forward]*5′-TTCAGCTACGAATCTCCGAC-3′*
[Reverse] *5′-ACCAGCATCTTCCTCAGCTT-3′*
SNAP25
[Forward]*5′-TCGTGTAGTGGACGAACGG-3′*
[Reverse] *5′-TCTCATTGCCCATATCCAGGG-3′*
SNX14
[Forward]*5′-AATCACCAACACGCAATTCA-3′*
[Reverse] *5′-TAGGCAACATAGCTCCCTCC-3′*
STX1A
[Forward]*5′-TAAAGAGCATCGAGCAGTCCA-3′*
[Reverse] *5′-GACATGACCTCCACAAACTTTCT-3′*
SNCA
[Forward]*5′-TGACGGGTGTGACAGCAGTAG-3′*
[Reverse]*5′-CAGTGGCTGCTGCAATGC-3′*
TNFA
[Forward]*5′-CAGAGGGCTGATTAGAGAGAGG-3′*
[Reverse] *5′-CTCATCTACTCCCAGGTCCTC-3′*

Supplementary Table 1: RT-PCR primers sequences

Supplementary Table 2

**Figure 1**	**Shapiro-Wilk test**		
**Panel B**	**W**	**p-value**	**normality result**
Time 0	0.901	0.4359	Yes
Ctrl	0.957	0.7601	Yes
EL 1t	0.9596	0.7763	Yes
**Panel C**	**W**	**p-value**	**normality result**
Ctrl	0.9316	0.6041	Yes
EL 1t	0.8938	0.4008	Yes
**Panel D**	**W**	**p-value**	**normality result**
Time 0	0.901	0.4359	Yes
Ctrl	0.9305	0.5974	Yes
EL 1t	0.8814	0.3444	Yes
EL 3t	0.9426	0.6705	Yes
**Panel E**	**W**	**p-value**	**normality result**
Ctrl	0.9523	0.7307	Yes
EL 1t	0.948	0.7035	Yes
EL 3t	0.9616	0.7887	Yes

**Figure 2**	**Shapiro-Wilk test**

Panel D	W	p-value	normality result
Ctrl	0.8558	0.2456	Yes
EL 1t	0.9469	0.6966	Yes

Panel E	W	p-value	normality result
Ctrl	0.9469	0.6966	Yes
EL 1t	0.8825	0.3493	Yes
EL 3t	0.9492	0.7112	Yes

Figure 6	Shapiro-Wilk test		
**Panel A**	**W**	**p-value**	**normality result**
Ctrl	0.8811	0.3141	Yes
EL 1t	0.9109	0.4731	Yes
**Panel B**	**W**	**p-value**	**normality result**
Ctrl	0.8811	0.3141	Yes
EL 1t	0.7960	0.0751	Yes
**Panel C**	**W**	**p-value**	**normality result**
Ctrl	0.9578	0.7651	Yes
EL 1t	0.9167	0.5186	Yes
**Panel D**	**W**	**p-value**	**normality result**
Ctrl	0.9624	0.7937	Yes
EL 1t	0.8798	0.3377	Yes
**Panel E**	**W**	**p-value**	**normality result**
Ctrl	0.8691	0.2227	Yes
EL 1t	0.8600	0.2281	Yes
EL 3t	0.9266	0.5731	Yes
**Panel F**	**W**	**p-value**	**normality result**
Ctrl	0.8811	0.3141	Yes
EL 1t	0.9166	0.5081	Yes
EL 3t	0.8182	0.1130	Yes
**Panel G**	**W**	**p-value**	**normality result**

Ctrl	0.9604	0.7812	Yes
EL 1t	0.9447	0.6833	Yes
EL 3t	0.9247	0.5633	Yes
**Panel H**	**W**	**p-value**	**normality result**
Ctrl	0.9298	0.593	Yes
EL 1t	0.896	0.4115	Yes
EL 3t	0.962	0.7915	Yes

**Figure 7**	**Shapiro-Wilk test**		
**Panel A**	**W**	**p-value**	**normality result**
Ctrl	0.8810	0.314	Yes
EL 1t	0.9737	0.8983	Yes
**Panel B**	**W**	**p-value**	**normality result**
Ctrl	0.8810	0.314	Yes
EL 1t	0.8429	0.1730	Yes
**Panel C**	**W**	**p-value**	**normality result**
Ctrl	0.8810	0.314	Yes
EL 1t	0.9591	0.8014	Yes
**Panel D**	**W**	**p-value**	**normality result**
Ctrl	0.8810	0.314	Yes
EL 1t	0.8925	0.3698	Yes
**Panel E**	**W**	**p-value**	**normality result**
Ctrl	0.9584	0.7687	Yes
EL 1t	0.8656	0.2808	Yes
**Panel F**	**W**	**p-value**	**normality result**
Ctrl	0.8581	0.2536	Yes
EL 1t	0.8907	0.3864	Yes
**Panel G**	**W**	**p-value**	**normality result**
Ctrl	0.8714	0.3032	Yes
EL 1t	0.9616	0.789	Yes
**Panel H**	**W**	**p-value**	**normality result**

Ctrl	0.8810	0.314	Yes
EL 1t	0.9209	0.5356	Yes
EL 3t	0.8474	0.1864	Yes
**Panel I**	**W**	**p-value**	**normality result**
Ctrl	0.8810	0.314	Yes
EL 1t	0.9351	0.6312	Yes
EL 3t	0.9655	0.8456	Yes
**Panel J**	**W**	**p-value**	**normality result**
Ctrl	0.8810	0.314	Yes
EL 1t	0.9126	0.4832	Yes
EL 3t	0.9032	0.4279	Yes
**Panel K**	**W**	**p-value**	**normality result**
Ctrl	0.8810	0.314	Yes
EL 1t	0.8719	0.2743	Yes
EL 3t	0.9233	0.5513	Yes
**Panel L**	**W**	**p-value**	**normality result**
Ctrl	0.8411	0.1988	Yes
EL 1t	0.9609	0.7843	Yes
EL 3t	0.9475	0.7002	Yes
**Panel M**	**W**	**p-value**	**normality result**
Ctrl	0.9538	0.7401	Yes
EL 1t	0.8927	0.3959	Yes
EL 3t	0.8707	0.3005	Yes
**Panel N**	**W**	**p-value**	**normality result**
Ctrl	0.9464	0.6937	Yes
EL 1t	0.9596	0.7765	Yes
EL 3t	0.8854	0.3624	Yes

**Figure 8**	**Shapiro-Wilk test**		
**Panel A**	**W**	**p-value**	**normality result**
Ctrl	0.8811	0.3141	Yes
EL 1t	0.8942	0.3787	Yes
**Panel B**	**W**	**p-value**	**normality result**

Ctrl	0.8811	0.3141	Yes
EL 1t	0.8797	0.3078	Yes
**Panel C**	**W**	**p-value**	**normality result**
Ctrl	0.8691	0.2227	Yes
EL 1t	0.8094	0.0964	Yes
EL 3t	0.9901	0.9800	Yes
**Panel D**	**W**	**p-value**	**normality result**
Ctrl	0.8691	0.2227	Yes
EL 1t	0.8410	0.1678	Yes
EL 3t	0.8773	0.2973	Yes

**Figure 9**	**Shapiro-Wilk test**		
**Panel A**	**W**	**p-value**	**normality result**
Ctrl	0.9020	0.4211	Yes
EL 1t	0.7915	0.0689	Yes
**Panel B**	**W**	**p-value**	**normality result**
Ctrl	0.9020	0.4211	Yes
EL 1t	0.9407	0.6711	Yes
**Panel C**	**W**	**p-value**	**normality result**
Ctrl	0.9020	0.4211	Yes
EL 1t	0.9032	0.4279	Yes
**Panel D**	**W**	**p-value**	**normality result**
Ctrl	0.9020	0.4211	Yes
EL 1t	0.8558	0.2135	Yes
**Panel E**	**W**	**p-value**	**normality result**
Ctrl	0.9020	0.4211	Yes
EL 1t	0.9318	0.6084	Yes
**Panel F**	**W**	**p-value**	**normality result**
Ctrl	0.9020	0.4211	Yes

EL 1t	0.9807	0.9386	Yes
EL 3t	0.8396	0.1638	Yes
**Panel G**	**W**	**p-value**	**normality result**
Ctrl	0.9020	0.4211	Yes
EL 1t	0.8266	0.1311	Yes
EL 3t	0.8804	0.3110	Yes
**Panel H**	**W**	**p-value**	**normality result**
Ctrl	0.9020	0.4211	Yes
EL 1t	0.7765	0.0514	Yes
EL 3t	0.9229	0.5485	Yes
**Panel I**	**W**	**p-value**	**normality result**
Ctrl	0.9020	0.4211	Yes
EL 1t	0.9074	0.4521	Yes
EL 3t	0.9479	0.7223	Yes
**Panel J**	**W**	**p-value**	**normality result**
Ctrl	0.9020	0.4211	Yes
EL 1t	0.9211	0.5371	Yes
EL 3t	0.9342	0.6255	Yes

**Figure 10**	**Shapiro-Wilk test**		
**Panel A**	**W**	**p-value**	**normality result**
Ctrl	0.8382	0.1902	Yes
EL 1t	0.9445	0.6820	Yes
**Panel B**	**W**	**p-value**	**normality result**
Ctrl	0.9628	0.7962	Yes
EL 1t	0.9641	0.8049	Yes
**Panel C**	**W**	**p-value**	**normality result**
Ctrl	0.8606	0.2624	Yes
EL 1t	0.8784	0.3318	Yes
**Panel D**	**W**	**p-value**	**normality result**
Ctrl	0.9447	0.6830	Yes
EL 1t	0.8773	0.3274	Yes

EL 3t	0.8949	0.4064	Yes
**Panel E**	**W**	**p-value**	**normality result**
Ctrl	0.8780	0.3304	Yes
EL 1t	0.9574	0.7626	Yes
EL 3t	0.9617	0.7894	Yes
**Panel F**	**W**	**p-value**	**normality result**
Ctrl	0.9557	0.7518	Yes
EL 1t	0.9261	0.5718	Yes
EL 3t	0.9603	0.7811	Yes

Supplementary Table 2: Results of the Shapiro-Wilk test for assessing the normality of the data distribution divided by Figures

Supplementary Table 3

Figure	T-Test			One-way ANOVA		
	t value	DF	p-value	F	DF	p-value
Fig. 1; panel B				10.57	11	0.0043
Fig. 1; panel C	0.6814	6	0.5211			
Fig. 1; panel D				61.66	15	<0.0001
Fig. 1; panel E				2.326	11	0.1534
Fig. 2; panel D	0.4528	6	0.6666			
Fig. 2; panel E				25.96	11	0.0002
Fig. 6; panel A	6.103	8	0.0003			
Fig. 6; panel B	4.921	8	0.0012			
Fig. 6; panel C	0.7802	6	0.4649			
Fig. 6; panel D	1.270	6	0.2512			
Fig. 6; panel E				27.34	14	<0.0001
Fig. 6; panel F				6.933	14	0.01
Fig. 6; panel G				4.891	11	0.0365
Fig. 6; panel H				6.524	11	0.0177
Fig. 7; panel A	1.138	8	0.2882			
Fig. 7; panel B	1.43	8	0.1905			
Fig. 7; panel C	1.016	8	0.3393			
Fig. 7; panel D	1.443	8	0.1869			
Fig. 7; panel E	1.137	6	0.2989			
Fig. 7; panel F	2.008	6	0.0914			
Fig. 7; panel G	0.795	6	0.4569			
Fig. 7; panel H				4.468	14	0.0355

Fig. 7; panel I				3.552	14	0.0614
Fig. 7; panel J				0.2885	14	0.7545
Fig. 7; panel K				15.74	14	0.0004
Fig. 7; panel L				10.11	11	0.005
Fig. 7; panel M				8.18	11	0.0095
Fig. 7; panel N				7.378	11	0.0127
Fig. 8; panel A	3.098	8	0.0147			
Fig. 8; panel B	3.253	8	0.0117			
Fig. 8; panel C				39.99	14	<0.0001
Fig. 8; panel D				6.101	14	0.0149
Fig. 9; panel A	1.97	8	0.0844			
Fig. 9; panel B	1.3	8	0.2298			
Fig. 9; panel C	0.2515	8	0.8078			
Fig. 9; panel D	1.004	8	0.3449			
Fig. 9; panel E	0.6249	8	0.5495			
Fig. 9; panel F				10.85	14	0.002
Fig. 9; panel G				23.64	14	<0.0001
Fig. 9; panel H				23.35	14	<0.0001
Fig. 9; panel I				40.35	14	<0.0001
Fig. 9; panel J				22.11	14	<0.0001
Fig. 10; panel A	1.557	6	0.1705			
Fig. 10; panel B	0.3107	6	0.7665			
Fig. 10; panel C	0.3352	6	0.7489			
Fig. 10; panel D				68.04	11	<0.0001
Fig. 10; panel E				15.16	11	0.0013
Fig. 10; panel F				35.88	11	<0.0001

Supplementary Table 3: Full statistical report of all the data included in the manuscript

Supplementary Figure 1

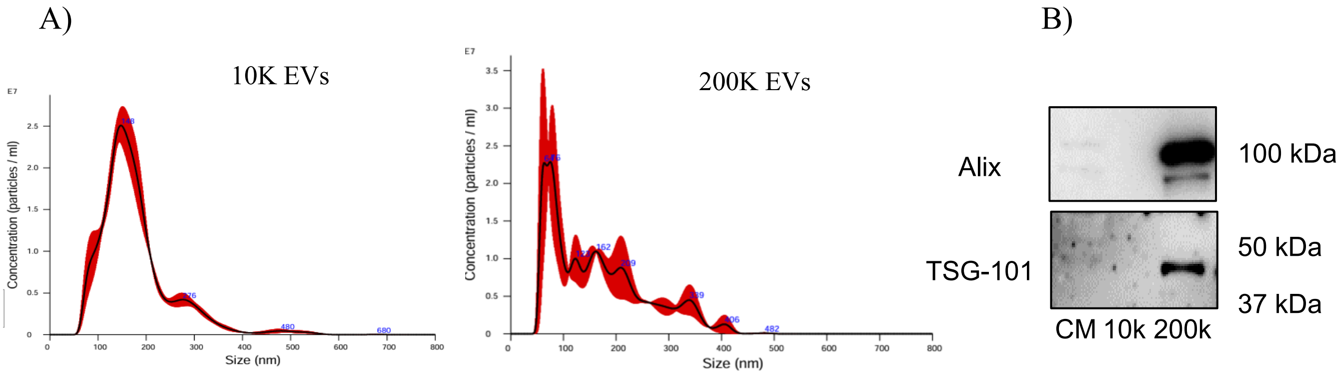

Supplementary Figure 1. Nanoparticle tracking analysis (NTA) of SH-SY5Y cell extracellular vesicles (EVs). (A) Size distribution of EVs obtained after 10000 x g centrifugation (10K), and after 200000 x g ultracentrifugation (200K). A representative sample is shown. (B) Enrichment of typical small EV markers (Alix and TSG-101) in 200K EVs, by Western blot analysis. CM, conditioned medium.

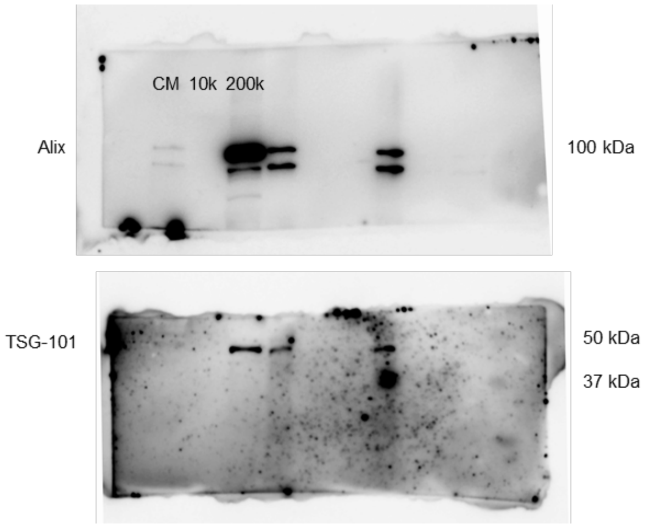

Original uncut western blots of Alix and TSG-101 proteins included in the Supplementary Figure 1 panel B.
